# Do predictors of adherence to pandemic guidelines change over time? A panel study of 22,000 UK adults during the COVID-19 pandemic

**DOI:** 10.1016/j.ypmed.2021.106713

**Published:** 2021-07-06

**Authors:** Liam Wright, Daisy Fancourt

**Affiliations:** a Department of Behavioural Science and Health, University College London, UK, WC1E 7HB; b Department of Epidemiology and Public Health, University College London, UK, WC1E 7HB

**Keywords:** COVID-19, compliance, non-pharmaceutical interventions

## Abstract

In the absence of a vaccine, governments have focused on behaviour change (e.g. social distancing and enhanced hygiene procedures) to tackle the COVID-19 pandemic. Existing research on the predictors of compliance with pandemic measures has often produced discrepant results. One explanation for this may be that the determinants of compliance are context specific. Understanding whether this is the case is important for designing public health messaging and for evaluating the generalisability of existing research. We used data from the UCL COVID-19 Social Study; a large weekly panel of UK adults from first five months of lockdown in the UK (n = 22,625). We tested whether the extent to which demographic, socio-economic position, personality traits, social and pro-social motivations, and the living environment predict compliance changed across the pandemic using multilevel regression modelling. Low compliance was strongly related to younger age and also to risk attitudes, empathic concern, and high income, among other factors. The size of some of these associations was larger in later months when less stringent lockdown and household mixing measures were in place. The results showed that compliance was lower and fell faster across some groups, suggesting the importance that public health communications adopt a plurality of messages to maximize broad adherence.

## Introduction

Governments have implemented a range of measures to tackle the COVID-19 pandemic. In the absence of a vaccine, measures have focused on reducing transmission of the virus through isolating those with diagnosed or suspected COVID-19, increasing ‘social distancing’ (e.g. ‘shelter-at-home’ orders, restricting non-essential travel and limiting groups gathering in public venues), and enhancing hygiene procedures (such as the wearing of face masks). These measures are effective ^1,2^ but require cooperation on the behalf of citizens. Ensuring high levels of compliance has been a challenge ^3–5^. Understanding the factors that determine compliance is vital for managing the pandemic.

There is a sizeable literature on the determinants of compliance with social distancing, hygiene, and quarantine rules, both from the COVID-19 pandemic and from previous epidemics ^6–8^. The literature highlights the importance of socio-economic and demographic characteristics (e.g. age ^6,7^, gender ^6,7,9^, education ^6,7^ and working status ^6,8,10^), personality traits (e.g. Big-5 traits ^11,12^ and self-efficacy ^6^), social and pro-social motivations (e.g. social norms ^13^, social capital ^14,15^ and empathic concern ^16^), and the lived environment (such as household overcrowding ^17^ and availability of green space ^18,19^), in predicting compliance levels. The results in this literature are not always consistent, however. For instance, younger people ^6^, males ^6,7^, and more extroverted individuals ^11,20^ are often found to have lower compliance than other individuals, but some studies show opposite or no effects ^6,21,22^.

One explanation for these discrepancies may be that the determinants of compliance are context-specific. For instance, self-efficacy beliefs, old age, and risk-preferences may change in importance as cases rise or as external restrictions on behavior change. This is consistent with models of human behavior such as the “COM-B” framework, which posits that capabilities, opportunities and motivations combine to determine behavior ^23^. For example, low self-efficacy may undermine psychological *capability* and risk-preferences and age may factor into non-compliance *motivations*, but strict lockdown may limit *opportunities* for non-compliance. Strict lockdown may thus represent a “strong situation” where opportunities for non-compliance are restricted and the information regarding desired behavior is clear, making personality and other characteristics less important for compliance behaviour ^11,24^.

This argument is supported by findings from the H1N1 pandemic that the predictors of compliance differed through time ^25^. It is also supported by findings from the COVID-19 pandemic that the Big-5 personality traits neuroticism and openness were more highly related to compliance with “shelter-at-home” guidelines in areas where less stringent measures were in place ^24^. Overall compliance levels do not remain stable across epidemics ^26–31^, which raises the possibility that the composition of noncomplying groups varies through time. Together, this suggests that existing COVID-19 studies – which have typically used cross-sectional data from the early months of the pandemic ^32–34^ – may not be generalisable across the full pandemic. Changes in the determinants of compliance may have implications for the targeting and phrasing of public health messaging and design of measures to maintain or improve compliance as pandemics proceed.

Therefore, in this study, we sought to test whether the association between factors relating to demographic and socio-economic characteristics, personality traits and pro-social motivations were more or less predictive of compliance with COVID-19 guidelines across five months of the pandemic in the UK. We used data from a weekly balanced panel of over 22,000 adults across the period 01 April – 31 August 2020, during which time the stringency of lockdown measures in the devolved nations of the UK changed ^35^. More detail on the change in government policy is provided in the methods section.

## Methods

### Participants

We used data from the COVID-19 Social Study; a large panel study of the psychological and social experiences of over 50,000 adults (aged 18+) in the UK during the COVID-19 pandemic. The study commenced on 21 March 2020 and involved online weekly data collection across the pandemic in the UK. The sampling is not random and therefore is not representative of the UK population, but it does contain a heterogeneous sample. The sample was recruited using three primary approaches. First, snowballing was used, including promoting the study through existing networks and mailing lists (including large databases of adults who had previously consented to be involved in health research across the UK), print and digital media coverage, and social media. Second, more targeted recruitment was undertaken focusing on (i) individuals from a low-income background, (ii) individuals with no or few educational qualifications, and (iii) individuals who were unemployed. Third, the study was promoted via partnerships with third sector organisations to vulnerable groups, including adults with pre-existing mental health conditions, older adults, carers, and people experiencing domestic violence or abuse. The study was approved by the UCL Research Ethics Committee [12467/005] and all participants gave informed consent. The study protocol and user guide (which includes full details on recruitment, retention, data cleaning, weighting and sample demographics) are available at https://github.com/UCL-BSH/CSSUserGuide.

For these analyses, we focused on participants with data collection in each month between 01 April – 31 August (n = 23,252, observations = 427,161). Recruitment into the study was ongoing across this period. This sample represents 38.8% of those who participated by 30 April 2020. We excluded participants with missing data on key demographic data that we use to construct survey weights (n = 627, observations = 11,480). We used complete case analysis as (a) there was only a small amount of item missingness in the study and (b) item missingness was dictated by not having data collection during limited periods in which particular questions were included in the survey, which suggests data are missing at random.

### Lockdown Measures

National lockdown was announced on 23 March 2020. Residents were required to stay at home unless purchasing essential items, exercising in nearby outdoor locations (at most once a day), or helping vulnerable individuals. Public venues were closed and businesses operated with strict social distancing guidelines or using remote working, where possible. Restrictions were gradually reduced from May 2020 with variation across the devolved nations in both the timing and extent of the reduction ^36^. Domestic travel limits were removed from 13 May in England but were relaxed later in Northern Ireland (26 June), Wales (6 July), and Scotland (7 July). Restrictions on indoor and outdoor household mixing were also gradually reduced (in June in England and Northern Ireland and July in Wales and Scotland), though local lockdowns were subsequently imposed in higher transmission areas of the UK from July. In the latter months, the UK government passed laws making face masks compulsory in public places and also began actively encouraging citizens to return to workplaces and to public venues – for instance, in August, running a subsidized meal scheme (“Eat Out to Help Out”) to support the restaurant sector. See Supplementary Figure 1.

### Measures

#### Compliance with COVID-19 Guidelines

Compliance with guidelines was measured weekly using a single-item measure: “Are you following the recommendations from authorities to prevent spread of Covid-19?”. The item was measured on a seven-point Likert scale (1 = “Not at all”, 7 = “Very much so”), and analysed as a continuous variable.

While this measure asks about compliance in general rather than referring to specific behaviours, it is related to items on specific compliance behaviours (mask wearing, social distancing, etc.) that have been collected at later time-points in the COVID-19 Social Study (see Supplementary Information).

#### Predictors of Compliance

To assess socioeconomic and demographic predictors of compliance, we included the demographic variables for country of residence, sex, ethnicity (White or Non-White) and age (grouped). We also included variables for socio-economic position (SEP): annual income (grouped), education level, employment status, household overcrowding (< 1 person per room, 1+ persons per room), and living arrangement (alone, with child; alone, no child; not alone, no child; not alone, with child). Each were measured at baseline data collection.

To assess predictors related to personality traits, we included Big Five personality traits (openness, conscientiousness, extraversion, agreeableness, and neuroticism)^37^, resilience ^38^, locus of control ^39^, optimism ^40^, and risk-taking ^41^. Big Five traits were measured at baseline data collection, while other traits were measured at during follow-up. More detail on the measurement of the variables in this analysis is included in the Supplementary Information.

To assess social and prosocial factors, we included measures of emotional and cognitive empathy, neighbourhood social capital, neighbourhood attachment, neighbourhood satisfaction ^42^, available neighbourhood space and neighbourhood crowding. Each of these variables were collected during follow-up. Neighbourhood social capital items referred to the period before COVID-19. Our hypothesis was that prosocial motivations would increase compliance, and that strong attachment or belonging to local neighbourhoods would increase these motivations as well as increasing the likelihood that individuals would be aware of – and pay attention to – injunctive social norms to comply with guidelines.

### Statistical Analysis

Our basic empirical strategy was to estimate 2-level multilevel models of the form:

$$\begin{array}{l} {\text{ Comply }}_{it}=\beta_{0i}+\beta_{1}\cdot {\text{ Month }}_{it}+\beta_{2}\cdot {\text{ Month }}_{it}\cdot {\text{ Predictor }}_{i}+\beta_{K}\cdot {\text{ Month }}_{it}\cdot X_{iK}+\varepsilon_{it}\\ \beta_{0i}=\beta_{0}+\mu_{i} \end{array}$$

Where *i* is an index of individuals and *t* is an index of time. *μ_i_* is a person-specific random error (i.e. a random intercept) and *ε_it_* is an observation-specific random error, both normally distributed. *Month_it_* is a categorical indicator of the month the data was collected in, *Predictor_i_* is the (time-invariant) predictor under study, and *X_iĸ_* is a set of control variables (defined below). Our interest is in the coefficients *β*_2_, which shows how the between-person association between the predictor and compliance differ across time.

For each predictor variable, we estimated two models: an unadjusted model with no control variables (except month), and an adjusted model that included (i) factors we identified as core confounders (including demographic and socio-economic variables as defined above, pre-existing psychiatric diagnosis, and Big-5 personality traits) and (ii) factors likely to account for differences in compliance behaviours (including self or family member “shielding” at any point due to pre-existing health conditions, whether the participant was remaining indoors for other pre-existing health reasons (e.g. physical disability), and the number of long-term physical health conditions (categorical: 0, 1, 2+). The definition of these measures in provided in the Supplementary Information. We used complete case data and survey weights when estimating models. Derivation of the survey weights is described in further detail in the Supplementary Information. For comparability, we report standardized coefficients.

An issue with restricting the analysis to individuals with data collection each month is that results may be biased by non-random attrition from the survey. In particular, individuals who report high compliance with COVID-19 guidelines are less likely to drop out (see Supplementary Information Figure S2), which may bias towards finding smaller associations. Consequently, as a sensitivity analysis, we repeat models using data from balanced panels with fewer months of data.

### Role of the Funding Source

The funders had no final role in the study design; in the collection, analysis and interpretation of data; in the writing of the report; or in the decision to submit the paper for publication. All researchers listed as authors are independent from the funders and all final decisions about the research were taken by the investigators and were unrestricted.

## Results

### Descriptive Statistics

Descriptive statistics for the sample are displayed in Table 1. Participants are disproportionately female, middle-aged (age 46-59), highly educated, white, and from Wales.

**Figure 1:**
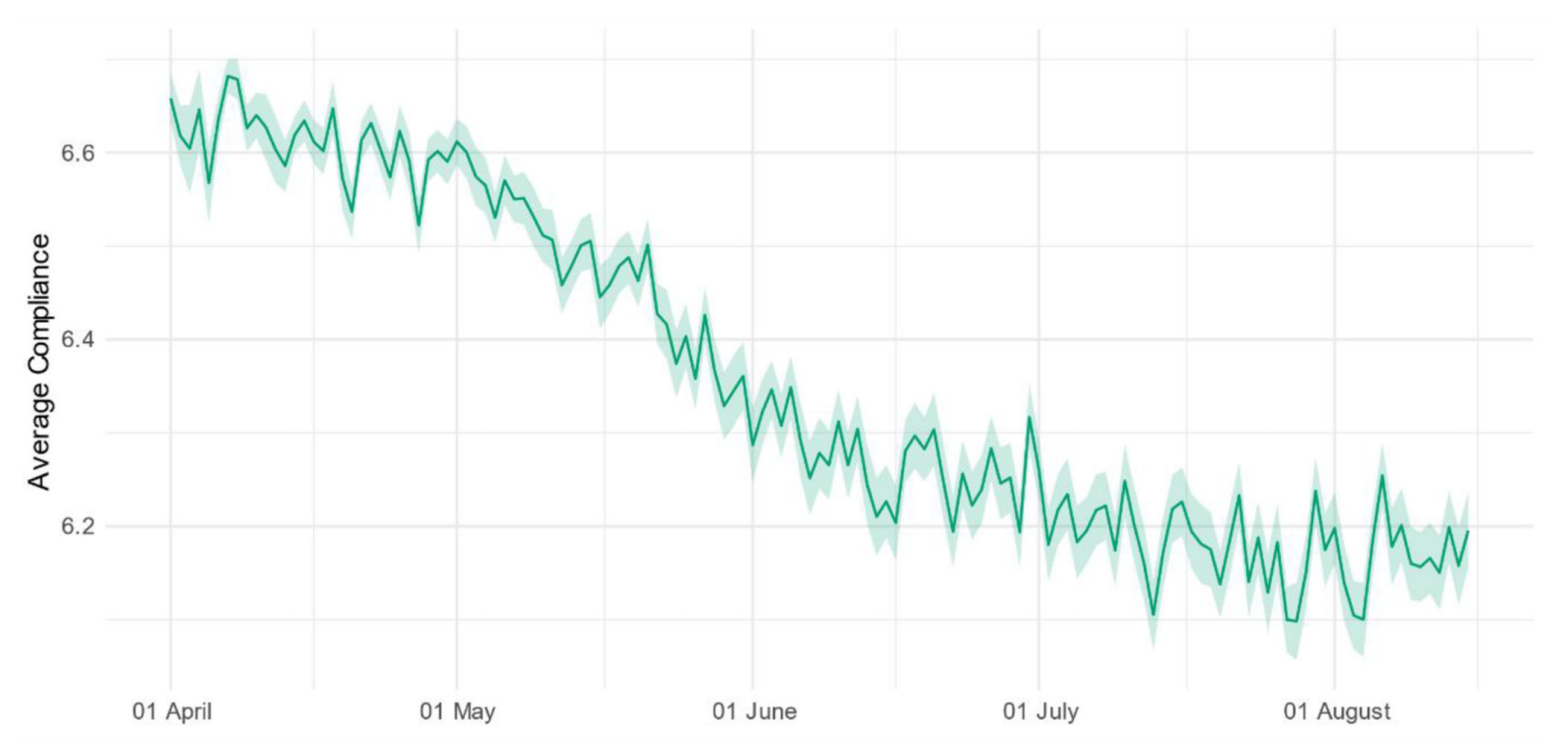
(Weighted) daily average compliance with COVID-19 guidelines (+ 95% CIs), 01 April – 15 August.

While compliance levels were high overall, there was a statistically significant decline in compliance with COVID-19 guidelines across the study period (Figure 1). When comparing descriptive statistics and compliance levels according to last month of data collection in the study, participants who were included in the main analysis (i.e. data collection up to August 2020) had higher and less steep drops in compliance, were older, less likely to be employed, and differed on several personality traits, including higher optimism and resilience and lower neuroticism and openness to experience (Table S1 and Supplementary Figure S2). Repeated cross-sectional data from YouGov ^27^ on the performance of specific preventative behaviours showed declines in avoiding crowds and social gatherings between April and August, while mask-wearing and hand-washing increased (Supplementary Figure S3).

### Demographics and Socio-Economic Position

**Figure 2:**
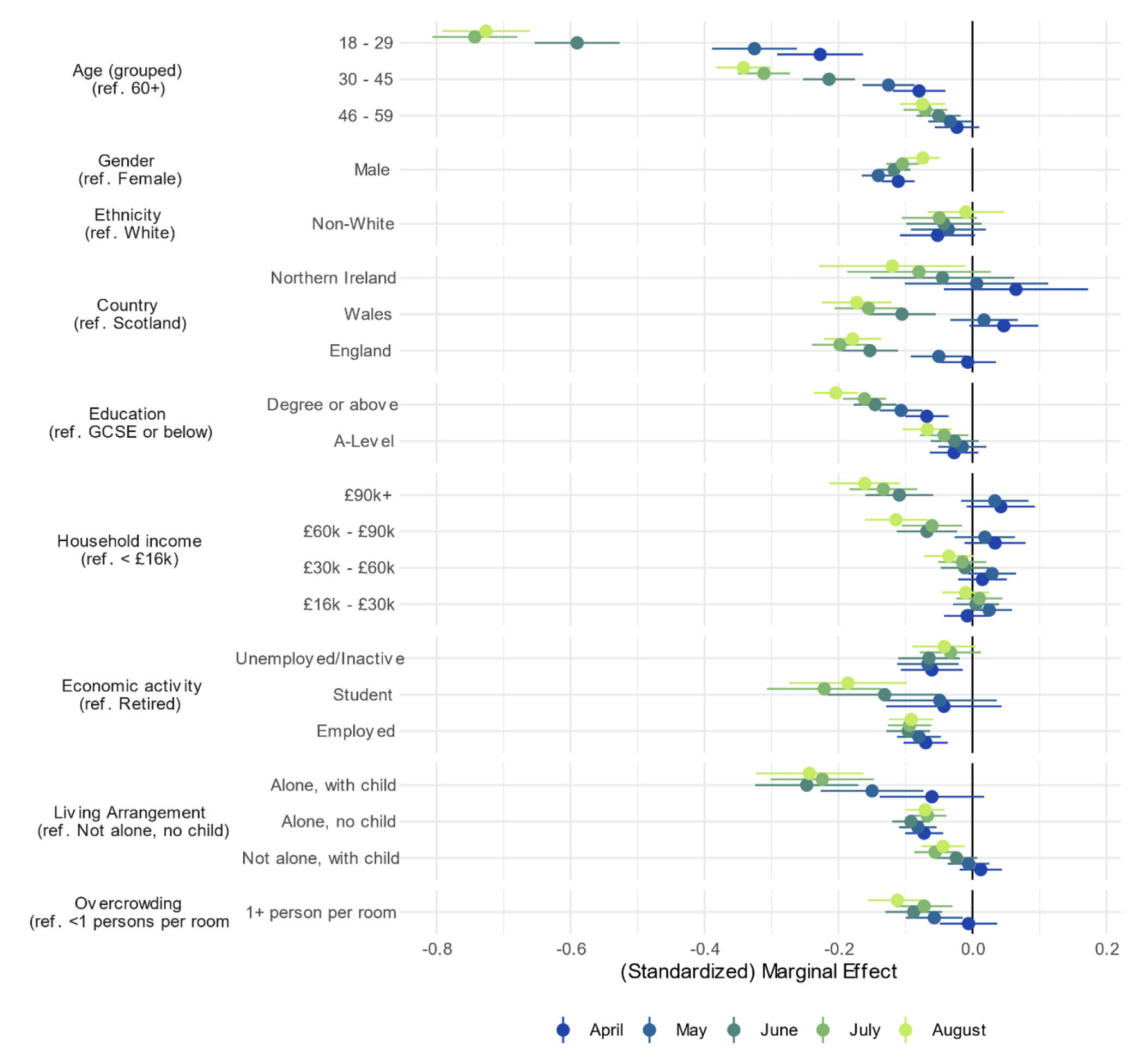
Association between socio-economic and demographic characteristics and compliance with COVID-19 guidelines by month, derived from mixed effects models. Models include adjustment for sex, age group, ethnicity, education, income group, employment status, country of residence, living arrangement, household overcrowding, whether the participant is shielding, diagnosed psychiatric condition, long-term physical health conditions, and Big-5 personality traits

Age-group, employment status, county of residence, income group, gender, living arrangement and household overcrowding were each related to compliance in multivariate models (Figure 2; bivariate results, which are substantively similar, are shown in Supplementary Figure S4.) Younger adults had lower levels of compliance than older adults in April and these age-related differences grew considerably as the pandemic continued. Females reported higher compliance than males, though there was little difference by month. There was little difference by ethnic group, but there was evidence that compliance was higher in Scotland than in England, to a somewhat greater extent in August than April/May. There was also an association between higher education and higher household income and lower compliance that grew across time. Students and individuals in employment had lower compliance levels than retired individuals. Comparing students and retirees, the difference was larger in later months. Adults living with a child or in overcrowded accommodation had lower compliance rates, with this increasing over the summer months. Effect sizes were generally small (< 0·25 SD), except for age in latter months: by August, 18-29 year olds had 0·72 SD (95% CI = 0·66, 0·79) lower compliance than participants aged 60+.

### Personality Traits

**Figure 3:**
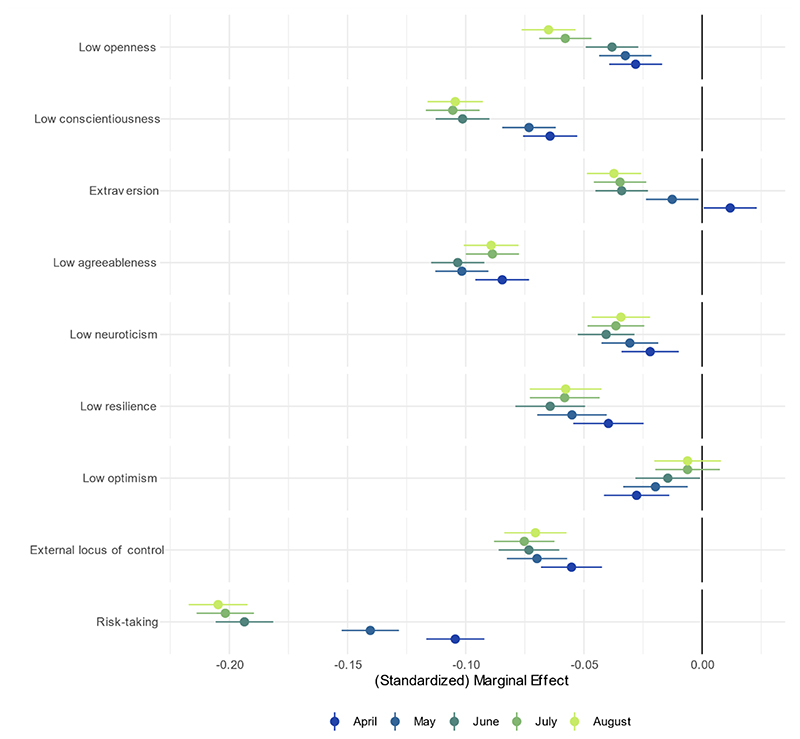
Association between personality traits and compliance with COVID-19 guidelines by month, derived from mixed effects models. Left panel: bivariate association. Models include adjustment for sex, age group, ethnicity, education, income group, employment status, country of residence, living arrangement, household overcrowding, whether the participant is shielding, diagnosed psychiatric condition, long-term physical health conditions, and Big-5 personality traits.

Each of the Big-5 traits, resilience, low optimism, locus of control, and risk tacking were related to compliance (Figure 3; note the different scale used for the x-axis). The strongest association was with risk taking, for which the association was almost twice as large in July/August than in April. Conscientiousness, openness to experience, and extraversion also become more highly related to compliance in later months, which low optimism became less strongly associated. Associations between compliance and agreeableness, resilience, locus of control and neuroticism were little changed. The bivariate results are substantively similar, except for extraversion, which was related to higher compliance (Supplementary Figure S5). Effect sizes were small (less than approximately 0·2 SD), in each case.

### Social and Prosocial Factors

**Figure 4:**
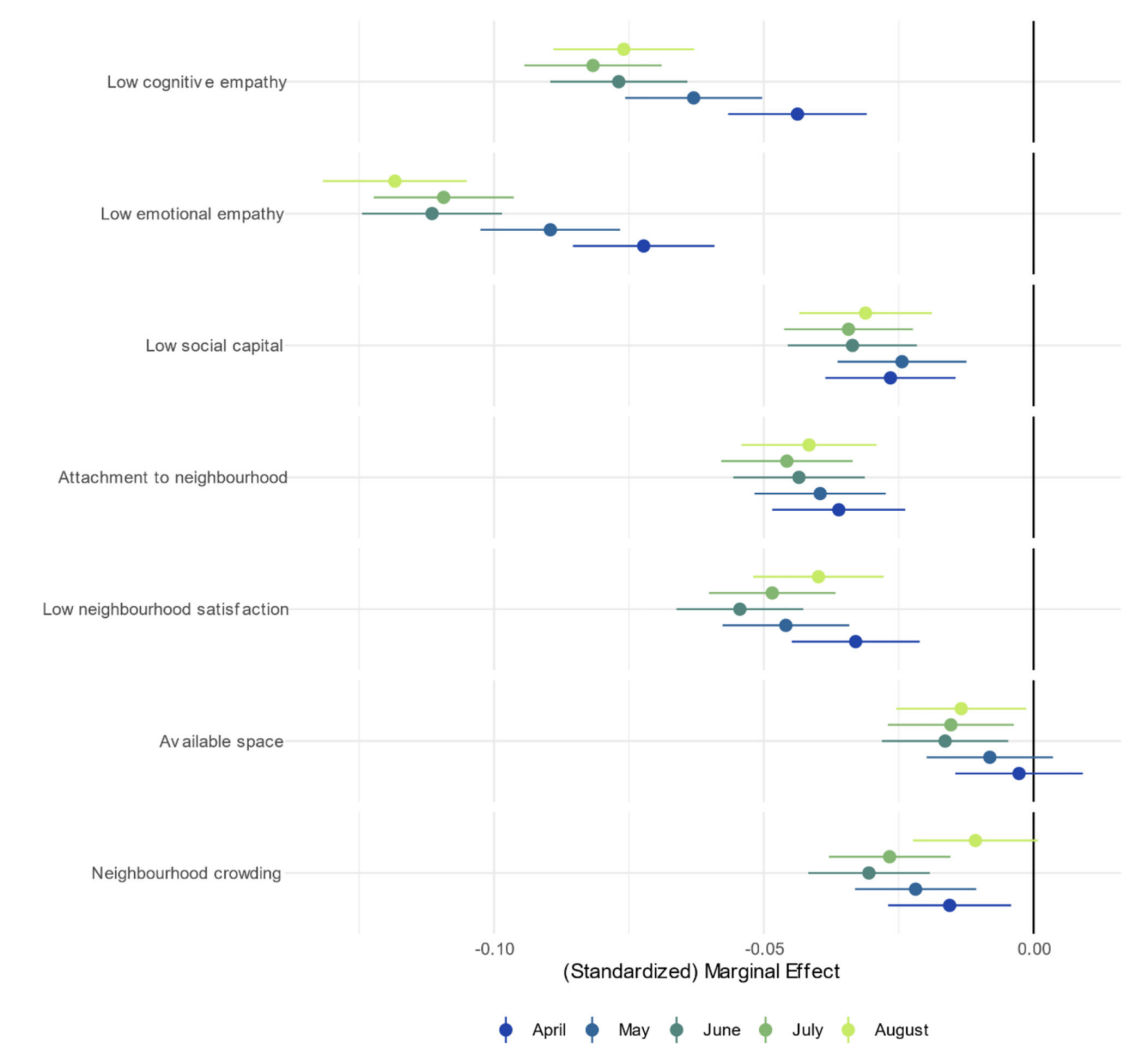
Association between prosocial motivations, neighbourhood factors and compliance with COVID-19 guidelines by month, derived from mixed effects models. Models include adjustment for sex, age group, ethnicity, education, income group, employment status, country of residence, living arrangement, household overcrowding, whether the participant is shielding, diagnosed psychiatric condition, long-term physical health conditions, and Big-5 personality traits.

Lower empathy, neighbourhood attachment and social capital were each related to lower compliance (Figure 4; note the different scale used for the x-axis). People with higher levels of cognitive or emotional empathy were more likely to continue complying in later months, while there was little change in associations for the other factors. Dissatisfaction with neighbourhood, available space and neighbourhood crowding were each related to lower compliance. The association between neighbourhood characteristics and compliance was broadly stable across time. Bivariate results are substantively similar, with the exception that there was no clear increase in the size of the association between low empathy and compliance in later months (Supplementary Figure S6). Effect sizes were small in each instance.

### Sensitivity Analysis

Point estimates were similar and results qualitatively the same when using balanced panels of sample members prior to August (Supplementary Figures S7-S12). This suggests that results are not driven by non-random attrition from the sample.

## Discussion

This study explored changes in the between-person predictors of compliance with COVID-19 guidelines in a longitudinal sample of UK adults across the first five months of social distancing and lockdown measures in the UK. Compliance was associated with major demographic and socio-economic characteristics, personality traits, prosocial motivations and neighbourhood characteristics. In general, effect sizes were small, with the exception of a strong association in later months with age. Specifically, decreases in average compliance levels over the period examined were more pronounced among young people (see Supplementary Figure S13), individuals low in conscientiousness and empathic concern, and individuals with risk-seeking attitudes. However, associations with compliance did not differ over the study period for all characteristics, with some (including agreeableness, neighbourhood attachment and neighbourhood characteristics) not changing in strength as predictors of compliance over time.

Some of the predictors of compliance identified in this study have been identified in previous studies, including low empathy, age, gender, and social capital ^6,11,43^. The finding that demographic characteristics (besides age) are not strongly related to compliance is also consistent with previous studies in the literature, which generally show that major individual demographic and socioeconomic characteristics and personality traits do not explain much of the between-person variation in compliance levels ^11,20,32^. However, previous literature has been cross-sectional and marred with inconsistencies in findings, and the results from this study help to explain why. Instead of being constant predictors of compliance over time, the differential strength of so many predictors of compliance over the five months of the study suggests that the determinants of compliance are context-specific. One explanation for these contextual differences is “situational strength”, whereby behaviour is less determined by personal characteristics in contexts where options are constrained and/or normative behaviour is clearly prescribed ^11,24,44^. In support of this theory, decreases in average compliance coincided with lockdown and social distancing restrictions becoming less stringent. Indeed, we found that compliance decreased fastest in England and Wales (where restrictions were eased fastest) compared to Scotland (where restrictions were kept more stringent for longer) ^36^. A further potential explanation for the differential strength of predictors is that as restrictions continued for longer, boredom increased and self-control depleted, meaning that individuals’ abilities to adhere to restrictive rules decreased and the predictive power of the studied factors became more evident ^45,46^. However, there were also other relevant changes over the period studied, which may have a bearing on results, including declining confidence in government ^3,47^ and changes in public health messaging (e.g. from “Stay at Home” to “Stay Alert”). Nevertheless, this study highlights the importance of considering individual traits as evolving predictors of compliance and, in particular, considering the role of context as a moderator of the relationship between traits and compliance.

The results have a number of policy implications. First, the findings suggest that compliance with pandemic control measures decreases as the stringency of measures is reduced. This highlights the importance of reinforcing messaging on compliance as measures are eased to avoid perceptions that remaining measures are somehow unnecessary. Second, the results suggest some individuals may not be responsive to specific types of communications. For example, speculatively, individuals with high risk-taking propensities may be less responsive to messages about personal risk but more responsive to prosocial messages about the impact of their risk-taking on others. Therefore, in seeking to maximize compliance amongst different groups, a plurality of communication approaches may be required, though this would need to be balanced against the possibility of “alert fatigue”^48^. The results also highlight why punitive measures for non-compliance may not be especially effective ^49,50^. For example, in September 2020, the UK government increased fines for those caught violating household mixing rules. Whilst such measures have the potential to reduce non-compliance among those for whom personal risk of infection or protecting others is not sufficient motivation, it risks reducing prosocial motivations ^51^ and highlighting the extent of norm violations, which, for some, could reduce compliance. Third, the finding that individuals living in overcrowded accommodation and neighbourhoods with little space had lower and faster decreasing compliance suggests that poor quality housing and crowded lived environments could exacerbate challenges for governments in tackling public health emergencies, over and above the greater risk of interpersonal transmission due to higher proximity ^52^. Beside specific policies such as opening green spaces to improve compliance ^18^, tackling social inequalities more broadly may have consequences not only for general public health but also for behavioural management during pandemics.

That said, we also found that individuals with higher incomes had higher initial compliance but faster decreases over time. It is possible that these individuals were able to maintain a strict compliance initially due to not facing any financial barriers such as an inability to pay bills that may have driven to rules being broken in a search for work ^53^. However, as the pandemic continued, it may be that greater wealth and a sense of privilege or a lack of financial fear over fines may have driven a more relaxed approach to compliance. Given research showing that the non-compliance of people in positions of power has a negative impact on societal trust and others’ compliance ^3^, this highlights the importance of the consistent application of pandemic rules amongst all groups.

This study had a number of limitations. Although we included a wide array of demographic and personality variables in our models, results may still have been explained by unobserved confounding. However, given that the strength of some of the factors studied here differed across the pandemic (e.g. risk taking), it is questionable how confounding factors could have generated these temporal patterns. Second, participants self-reported their compliance with measures. Results may, therefore, have been biased due to social desirability concerns and due to differences in knowledge or interpretation of the questionnaire item. People’s understanding of compliance may also have differed over time, particular as rules changed. We lacked detail on the specific rules – such as mask-wearing, social distancing or hand-washing – that individuals were breaking when reporting lower compliance. Some of the factors studied here may be important for some behaviours rather than others – for instance, extraversion is more plausibly related to violating social distancing rules than non-mask wearing. Further, bias may have arisen through the specifics of the guidelines differing through time. Notably, rules on mask wearing were introduced in the latter part of the study. As mask wearing is one of the most complied with behaviours (see Supplementary Figure S3), the introduction of rules regarding masks may have reduced between-person differences in the latter stages of the study period: changes in particular behaviours according to person characteristics may be more pronounced than estimated here. Future studies are encouraged that explore specific compliance behaviours in more detail.

A third limitation is that we treated the predictors as fixed, but they may have varied over time – for instance, individual’s empathy may have changed across the pandemic. This may have explained some of the time varying associations, though we note that associations with compliance were not always strongest in the month the predictors were measured in (see, for instance, Big 5 personality trait results). A final issue is the use of a non-random sample. While the sample was heterogeneous and we included population weights in models, the data were from a study set-up explicitly to research COVID-19. It is likely that individuals who participated (and continued to participate) in the study had a higher interest in helping tackle the pandemic than the general population at large. This interest may manifest as a higher propensity to comply with guidelines. Further, selection biases are likely to have arisen due to different modes of recruitment into the survey. Therefore, the relationships identified here may be biased estimates of the actual predictive effects of some of these factors.

## Conclusion

This study demonstrated that the importance of many factors in predicting compliance has differed across the pandemic and is therefore context specific. Further, the results are in line with theories of “situational strength” where individual characteristics are more important where behaviour is less constrained. This highlights the need to account for context when studying compliance and to study predictors of compliance as evolving factors. For policy makers and public health professionals, the results suggest that if we want to maintain good compliance or increase compliance, two key things are needed. First, multiple messages are required to target different groups given capabilities and motivations will vary substantially between different demographic groups. Second, these messages will need to evolve across pandemics as the context and behavioural opportunities for individuals change.

## Supplementary Material

Supplementary Information

## Figures and Tables

**Table 1:** Descriptive Statistics

	Variable	Unweighted	Weighted
	n	22,625	22,625
Age (grouped)	60+	10,035 (44.35%)	11,679.69 (51.62%)
	46 – 59	7,144 (31.58%)	5,799.04 (25.63%)
	30 – 45	4,521 (19.98%)	3,632.15 (16.05%)
	18 – 29	925 (4.09%)	1,514.13 (6.69%)
Gender	Female	16,741 (73.99%)	11,111.33 (49.11%)
	Male	5,884 (26.01%)	11,513.67 (50.89%)
Ethnicity	White	21,882 (96.72%)	21,116.39 (93.33%)
	Non-White	743 (3.28%)	1,508.61 (6.67%)
Country	Scotland	1,441 (6.37%)	1,822.22 (8.05%)
	England	17,551 (77.57%)	18,624.75 (82.32%)
	Wales	3,413 (15.09%)	1,742.84 (7.7%)
	Northern Ireland	220 (0.97%)	435.18 (1.92%)
Education	GCSE or below	3,230 (14.28%)	7,494.49 (33.12%)
	A-Level	3,908 (17.27%)	7,271.22 (32.14%)
	Degree or above	15,487 (68.45%)	7,859.29 (34.74%)
Household income	< £16k	3,106 (15.27%)	4,099.51 (20.3%)
	£16k – £30k	5,417 (26.63%)	6,271.51 (31.06%)
	£30k – £60k	7,116 (34.98%)	6,394.21 (31.67%)
	£60k – £90k	2,823 (13.88%)	2,124.96 (10.52%)
	£90k+	1,881 (9.25%)	1,300.31 (6.44%)
Economic activity	Retired	7,554 (33.39%)	8,618.23 (38.09%)
	Employed	12,417 (54.88%)	10,677.94 (47.2%)
	Student	477 (2.11%)	785.86 (3.47%)
	Unemployed/Inactive	2,177 (9.62%)	2,542.97 (11.24%)
Living Arrangement	Not alone, no child	13,299 (58.78%)	13,987.61 (61.82%)
	Not alone, with child	3,891 (17.2%)	3,280.05 (14.5%)
	Alone, no child	4,990 (22.06%)	5,014.07 (22.16%)
	Alone, with child	445 (1.97%)	343.27 (1.52%)
Overcrowding	<1 persons per room	21,085 (93.19%)	20,616.22 (91.12%)
	1+ person per room	1,540 (6.81%)	2,008.78 (8.88%)
	Low openness	8.68 (3.24)	9.17 (3.25)
	Low conscientiousness	7.93 (2.89)	8.14 (2.94)
	Extraversion	12.79 (4.27)	12.59 (4.25)
	Low agreeableness	8.41 (3.01)	8.58 (3.09)
	Low neuroticism	13.05 (4.25)	13.2 (4.33)
	Low resilience	15.58 (5.15)	15.35 (5.24)
	Low optimism	16.14 (4.68)	16.62 (4.7)
	External locus of control	12.21 (2.63)	12.47 (2.69)
	Risk-taking	4.37 (2.34)	4.35 (2.42)
	Low cognitive empathy	9.28 (4.83)	10.01 (4.99)
	Low emotional empathy	7.27 (4.65)	8 (4.82)
	Low social capital	12.97 (3.46)	13.15 (3.59)
	Attachment to neighbourhood	7.09 (3.23)	7.24 (3.34)
	Low neighbourhood satisfaction	1.9 (0.92)	1.97 (0.94)
	Available space	3.58 (1.13)	3.65 (1.17)
	Neighbourhood crowding	4.96 (1.85)	5.01 (1.83)
Shielding (pre-existing condition)	No	18,024 (79.66%)	17,263.31 (76.3%)
	Yes	4,601 (20.34%)	5,361.69 (23.7%)
Shielding (family member)	No	19,678 (86.97%)	19,437.56 (85.91%)
	Yes	2,947 (13.03%)	3,187.44 (14.09%)
Home for other reason	No	21,671 (95.78%)	21,419.2 (94.67%)
	Yes	954 (4.22%)	1,205.8 (5.33%)
Psychiatric condition	No	19,156 (84.67%)	19,203.27 (84.88%)
	Yes	3,469 (15.33%)	3,421.73 (15.12%)
Long-Term Conditions	0	12,697 (56.12%)	11,660.97 (51.54%)
	1	6,342 (28.03%)	6,493.88 (28.7%)
	2+	3,586 (15.85%)	4,470.15 (19.76%)
