# Supplementary material for: Do predictors of adherence to pandemic guidelines change over time? A panel study of 22,000 UK adults during the COVID-19 pandemic
Source: Prev Med. Author manuscript; Available in PMC 2021 Aug 3. (PMC8259055; doi:10.1016/j.ypmed.2021.106713)
Supplement: Supplementary Information [file EMS130511-supplement-Supplementary_Information.docx]

# SUPPLEMENTARY MATERIAL

## MEASURES

### Demographics and socio-economic position

We included demographic variables for country of residence (England, Scotland, Wales, Northern Ireland), sex (male or female), ethnicity (White or Non-White) and age (grouped: 18-29, 30-45, 46-59, 60+). We also included variables for socio-economic position (SEP): annual income (grouped: < £16k, £16k - £30k, £30k - £60k, £60k - £90k, £90k +), education level (higher education, further education, secondary education or below), employment status (employed, student, unemployed/inactive, retired), household overcrowding (< 1 person per room, 1+ persons per room), and living arrangement (alone, with adult but no child, with child). Each were measured at baseline interview.

### Personality traits

Personality was measured at baseline interview using the Big Five Inventory (BFI-2; Soto & John, 2017), which measures five domains and 15 facets: openness (intellectual curiosity, aesthetic sensitivity, and creative imagination), conscientiousness (organisation, productiveness, and responsibility), extraversion (sociability, assertiveness, and energy level), agreeableness (compassion, respectfulness, and trust) and neuroticism (anxiety, depression, and emotional volatility). Each item was scored on a 5-point scale (1 = “strongly disagree”, 5 = “strongly agree”). We use the sum Likert score for each domain (range 3 - 15). As our interest is in the predictors of low compliance, in line with the results of previous studies (Brouard et al., 2020; Clark et al., 2020; Zajenkowski et al., 2020), we reverse coded items related to openness, conscientiousness, agreeableness and neuroticism so that high values indicate low levels of the trait.

Resilience was assessed between 14 – 21 May with the 6-item Brief Resilience Scale (Smith et al., 2008), a widely used measure of individuals’ ability to recover from stress. Items are rated on a five-point scale (1 = “strongly disagree”, 5 = “strongly agree”). We used the sum Likert score, with items coded such that higher scores indicate lower resilience (range 6 - 30).

Locus of control was measured between 04 – 11 June using the 6-item Locus of Control Scale developed by the University of Washington Beyond High School Project (Hirschman & Almgren, 2012), and captures generalized expectancies about whether individuals can (internal) or cannot (external) control events and outcomes in their lives. Responses were rated on a four-point scale ranging from (1 = “strongly agree”, 4 = “strongly disagree”). We used the sum score of responses, with internal worded items coded so higher values indicate more external locus of control.

Optimism was collected between 04 – 11 June using the 10-item Life Orientation Test-Revised (LOT-R; Scheier et al., 1994). Items are rated on a five-point scale ranging from “strongly disagree” to “strongly agree”. We used the sum Likert score, coding items such that higher scores indicate lower optimism (range 6 - 30).

Risk-taking was measured between 23-30 July with one item from the Dohmen Risk Taking Scale (Dohmen et al., 2011). Respondents rated the extent to which they generally see themselves as a person who is fully prepared to take risks, rated on an 11-point scale (0 = “not at all willing to take risks” to 10 = “very willing to take risks”).

### Social and Prosocial Factors

We included several measures of pro-social motivations: empathy and social capital. Neighbourhood social capital before COVID-19 was measured between 09-16 July by combining four items from the social cohesion subscale of the Neighbourhood Scales (Mujahid et al., 2007) and a further item, “Before COVID-19, this was a close-knit neighbourhood”. Items were rated on a five-point scale (1 = “strongly disagree”, 5 = “strongly agree”), with items reflecting beliefs about trust, shared values, and willingness to help others. We reverse-coded items and used the sum score of items on social capital prior to COVID-19 (range 5 – 25). Higher values indicating lower social capital.

Empathy was assessed between 11-18 July using subscales for empathic concern and perspective-taking from the Interpersonal Reactivity Index (IRI; Davis, 1983). (Subscales for fantasy and personal distress were not administered.) Empathic concern (also known as emotional empathy) consists of 7 items and captures feelings of warmth, concern, and compassion for others. Perspective-taking (also known as cognitive empathy) assesses efforts to adopt the perspectives of others (7 items). Items were rated on a five-point scale ranging (1 = “does not describe me well”, 5 = “describes me very well”). We used the sum Likert score for each domain, coding items such that higher scores indicate lower empathy (range 0 - 28).

We also assessed four aspects of individuals’ neighbourhoods, each measured between 09-16 July. Neighbourhood attachment was assessed with three five-point Likert items on whether the participant feels their neighbourhood is their home (1 = “just a place to live”, 5 = “home”), their attachment to their neighbourhood (1 = “no attachment”, 5 = “strong attachment”) and feelings of belonging in their community (1 = “don’t belong at all”, 5 = “belong strongly”). We used the sum Likert score (range 3-15). We reverse-code items so that higher scores indicate lower attachment.

Neighbourhood satisfaction was assessed with a five-point Likert item (1 = “very dissatisfied”, 5 = “very satisfied”). We reverse code this so that higher scores indicate lower satisfaction. Neighbourhood space was measured using three items on satisfaction with neighbourhood walkability, usable green space, and presence of trees. Each was measure on a three-point scale (1 = “dissatisfied”, 2 = “neither satisfied nor dissatisfied”, 3 = “satisfied”), which we reverse coded and summed into a single score (range 3 – 9). Neighbourhood crowding was measured using three items on satisfaction with neighbourhood traffic density, noise, and levels of crowding. Each was measured on a three-point scale (categories as above), which we reverse coded and summed into a single score (range 3 – 9). The items used to operationalize neighbourhood attachment, crowding and space were constructed for the COVID-19 Social Study. The neighbourhood satisfaction item has been used previously by Peck (1981).

### Covariates

In regression models, we also included adjustment for pre-existing psychiatric diagnosis, shielding due to (own or family) pre-existing health conditions, and number of long-term physical health conditions (categorical: 0, 1, 2+).

Long-term physical health conditions (0, 1, 2+) was measured using a multiple-choice question on medical conditions. Included conditions were high blood pressure, diabetes, heart disease, lung disease, cancer, any other clinically-diagnosed chronic physical health conditions, or any disability. Psychiatric diagnosis (yes, no) was with the same multiple choice question using items on clinically diagnosed depression, clinically diagnosed anxiety, and any other clinically diagnosed mental health problem. The data was collected at baseline interview.

We measured shielding with three separate variables on not leaving home due to being high risk, due to a family member being high risk, and due to other reasons not related to COVID-19 (e.g. maternity leave). Survey items on shielding were included in all data collections from 23 March – 04 July 2020. We defined a person as shielding if they stated shielding at any data collection.

### Timeline of Data Collection

| Data Collection | Measure |
| --- | --- |
| Baseline | Country of residence, ethnicity, age, annual income, education level, employment status, household overcrowding, living arrangement, Big Five personality traits |
| 14 – 21 May | Resilience |
| 04 – 11 June | Locus of Control; optimism; |
| 09 – 16 July | Neighbourhood social capital; neighbourhood attachment; neighbourhood satisfaction; neighbourhood crowding |
| 11 – 18 July | Empathy |
| 23 - 30 July | Risk taking |

## ASSOCIATION BETWEEN DEPENDENT VARIABLE AND COMPLIANCE BEHAVIOURS

Between 17 November – 23 December 2020, the COVID-19 Social Study included six items on self-reported compliance behaviour:

| In the last 7 days, to what extent have you been following the behaviours below? |
| --- |
| 1. Washing your hands thoroughly with soap and water or using hand sanitising gel after any possible contact with other people outside of your household or shared surfaces 2. Wearing a face mask or other face covering where it is currently recommended 3. Maintaining the recommended distance from people not in your household/bubble 4. Meeting up with MORE THAN the recommended number of people from other households OUTDOORS 5. Meeting up with MORE THAN the recommended number of people from other households INDOORS 6. Following other rules relevant to the tier or level of lockdown currently active in your area |

The response categories were: never, rarely, occasionally, frequently, always, not applicable. Note, items on household mixing were phrased such that higher scores indicated lower compliance.

The item on self-reported compliance with guidelines used in the present study was also collected between 17 November – 23 December, offering an opportunity to explore whether the item was associated with specific compliance behaviours. Figure S14 displays distributions of responses to each behaviour measure according to self-report compliance with guidelines. For each behaviour, higher self-reported compliance with guidelines is associated with higher compliance with the given behaviour. A regression of self-reported compliance on the six behaviour variables, with each inputted as a categorical variable, had an R^2^ value of 0.47.

The self-reported compliance item was also associated with two factors extracted from an exploratory factor analysis (EFA) of the compliance behaviour measures. In the EFA, we used polychoric correlations, promax rotation. and treated the “not applicable” response category as missing. Two factors had an eigenvalue greater than 1. The self-reported compliance item was correlated with the first factor (which loaded onto Items 1-3 and 6) with ρ = 0.64 and was correlated with the second factor (which loaded onto Items 4-5) with ρ = 0.36.

From 17 June 2020, each wave of the COVID-19 Social Study also included an item on social distancing:

| When you go out or meet with others have you been maintaining social distancing?”). |
| --- |
| 1. Not applicable - I have not met with others or left my home in the last week 2. Yes, completely 3. Yes, to a large extent 4. Not always 5. Not at all |

To test whether responses and trends in this item were correlated with the self-reported compliance with guidelines measures, we estimated growth curve models for each variables using data from participants in the COVID-19 Social Study with 3+ waves of data between 17 June 2020 – 17 May 2021. (For consistency, we reverse code the social distancing item so high scores indicate greater compliance.) We modelled time trends with natural splines with degrees of freedom 3, including random intercepts and slopes. The results are displayed in Figure S15. The predicted regression lines are almost perfectly correlated with ρ = 0.96.

Extracting the predicted trajectories for each participant, there is a high correlation between estimated *individual* trajectories in the social distancing and self-report compliance measures (median correlation ρ = 0.88). The correlation between the individual trajectories is itself not highly correlated with the continuous covariates used in the main analysis (| ρ | < 0.02 in each case).

## FIGURES


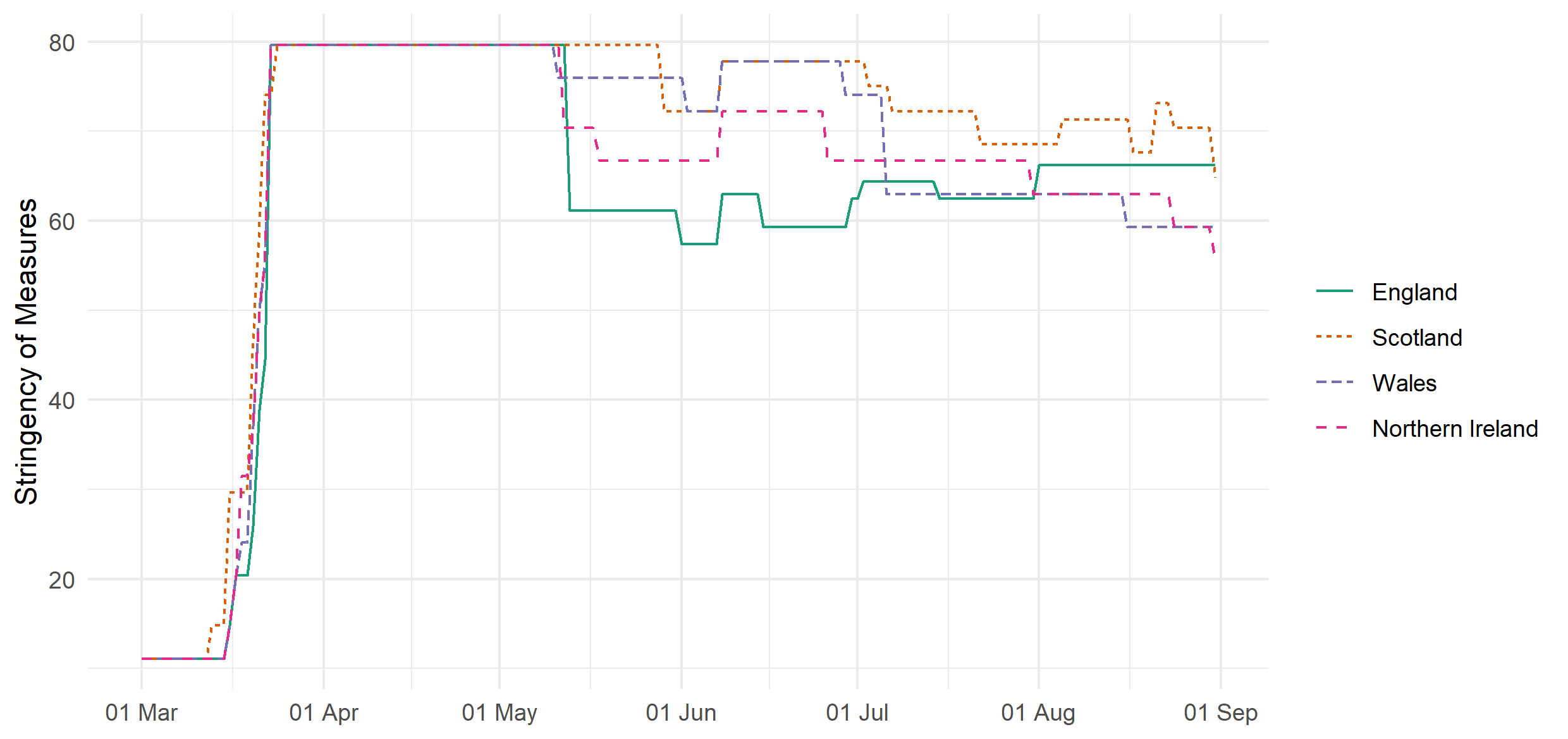


**Figure S1:** Stringency of UK government COVID-19 related measure, 02 January – 31 July. Source: Oxford COVID-19 Government Response Tracker (Hale et al., 2020).


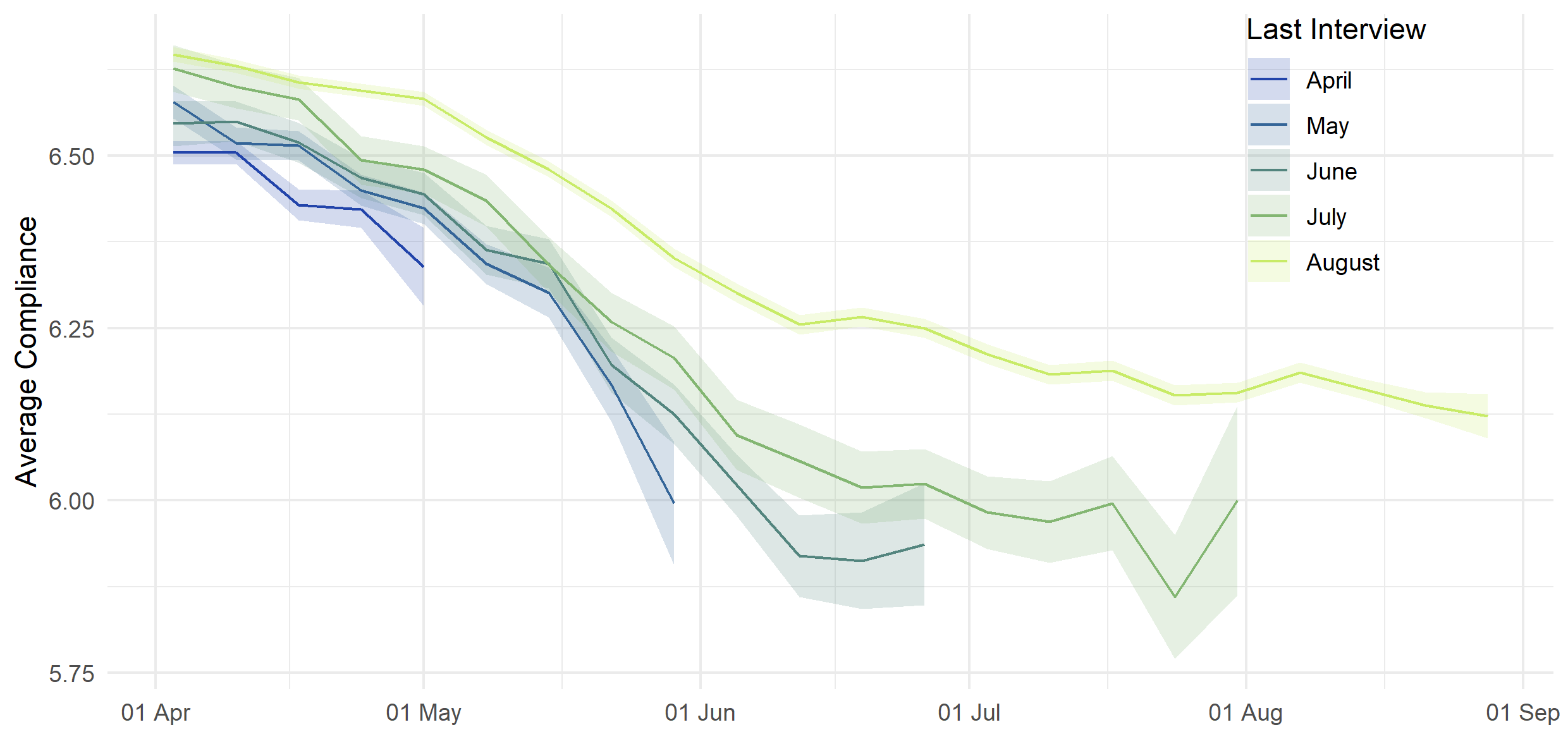


**Figure S2:** (Weighted) weekly average compliance by month of last interview. Participants with last interview in August were included in the main analysis.


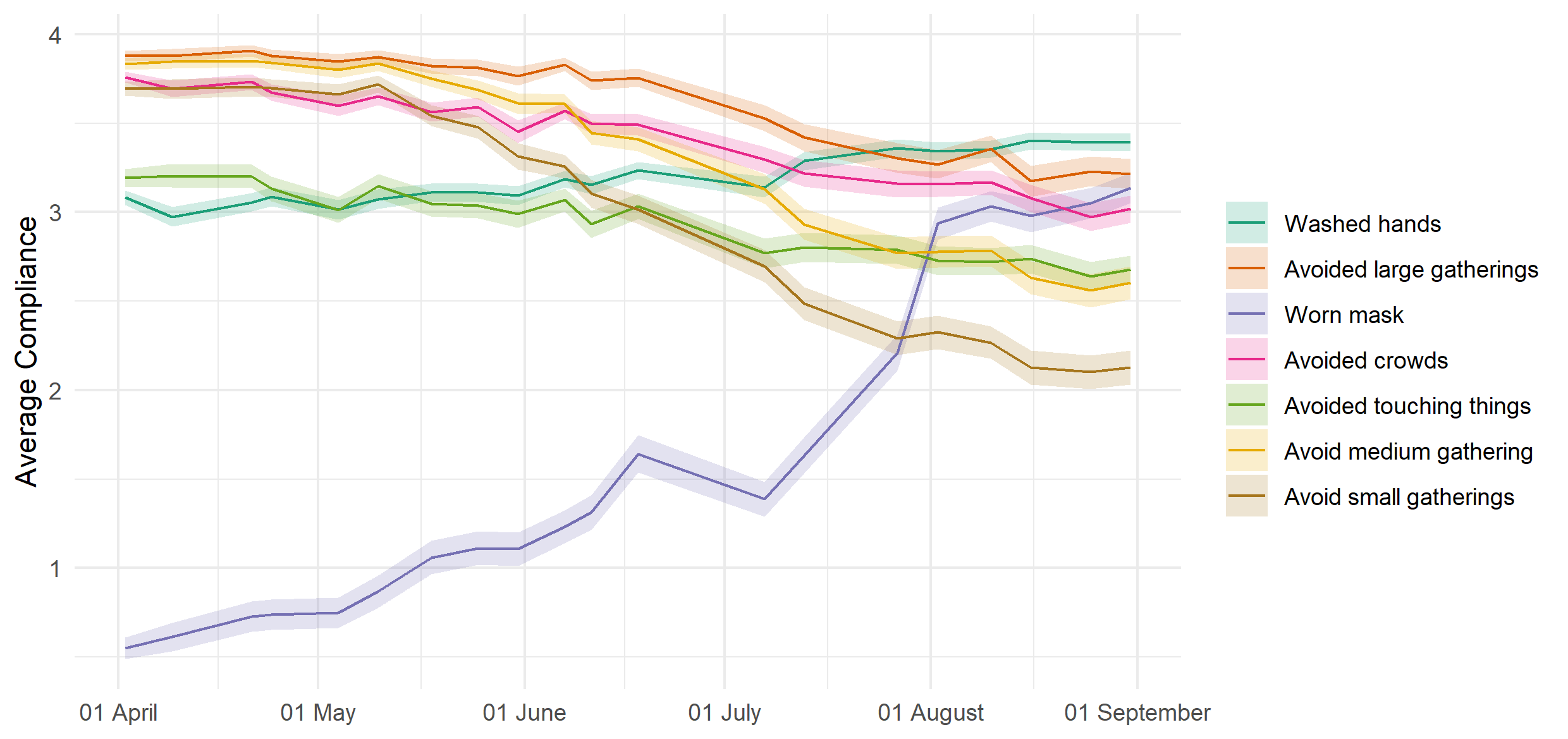


**Figure S3:** (Weighted) weekly average compliance with recommended preventative behaviours in the UK, 01 April – 31 August. Source: YouGov COVID-19 Public Monitor (YouGov, 2021).


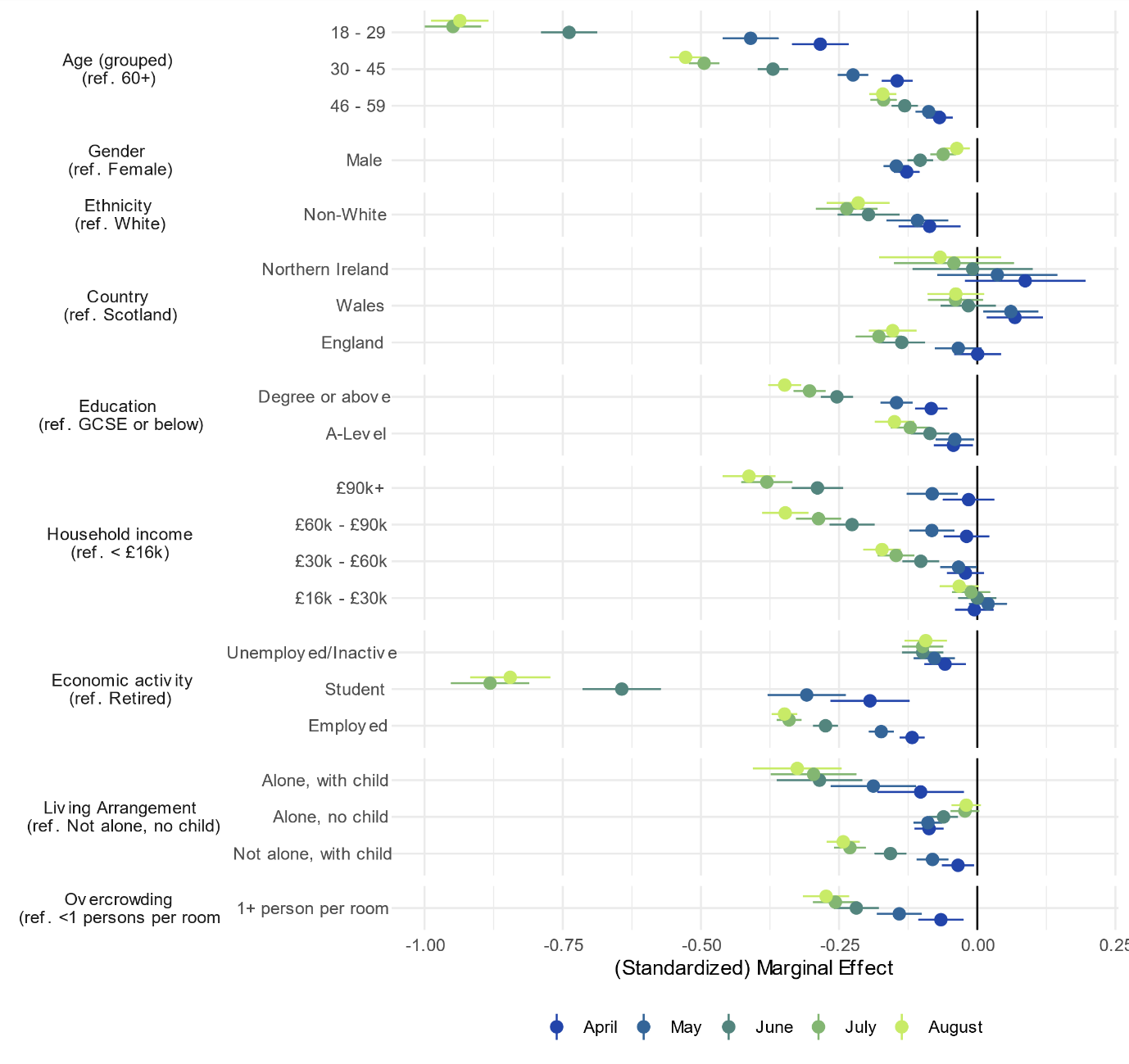


**Figure S4:** Association between demographic and socio-economic characteristics and compliance with COVID-19 guidelines by month, derived from mixed effects models. Bivariate associations.


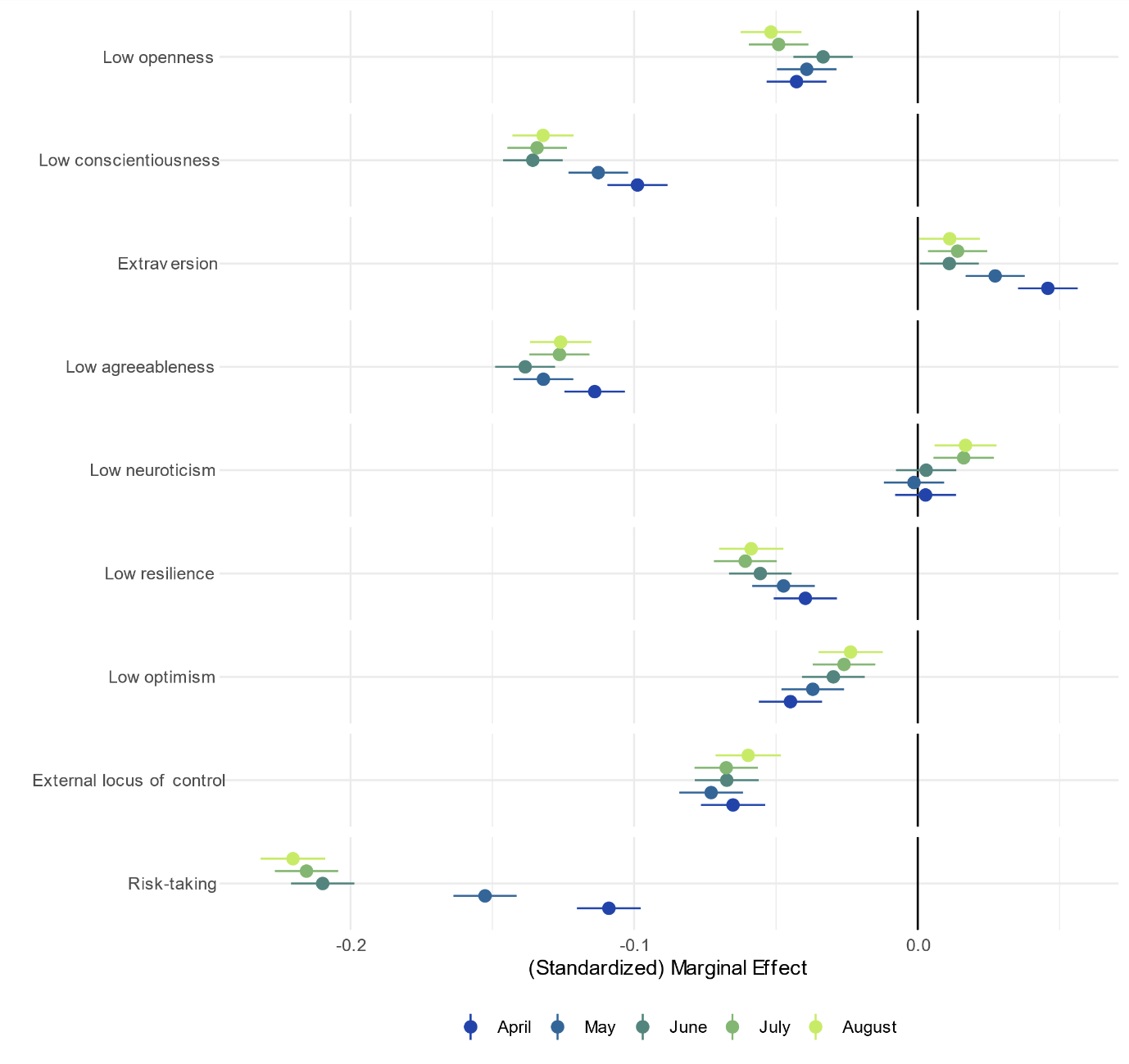


**Figure S5:** Association between personality traits and compliance with COVID-19 guidelines by month, derived from mixed effects models. Bivariate associations.


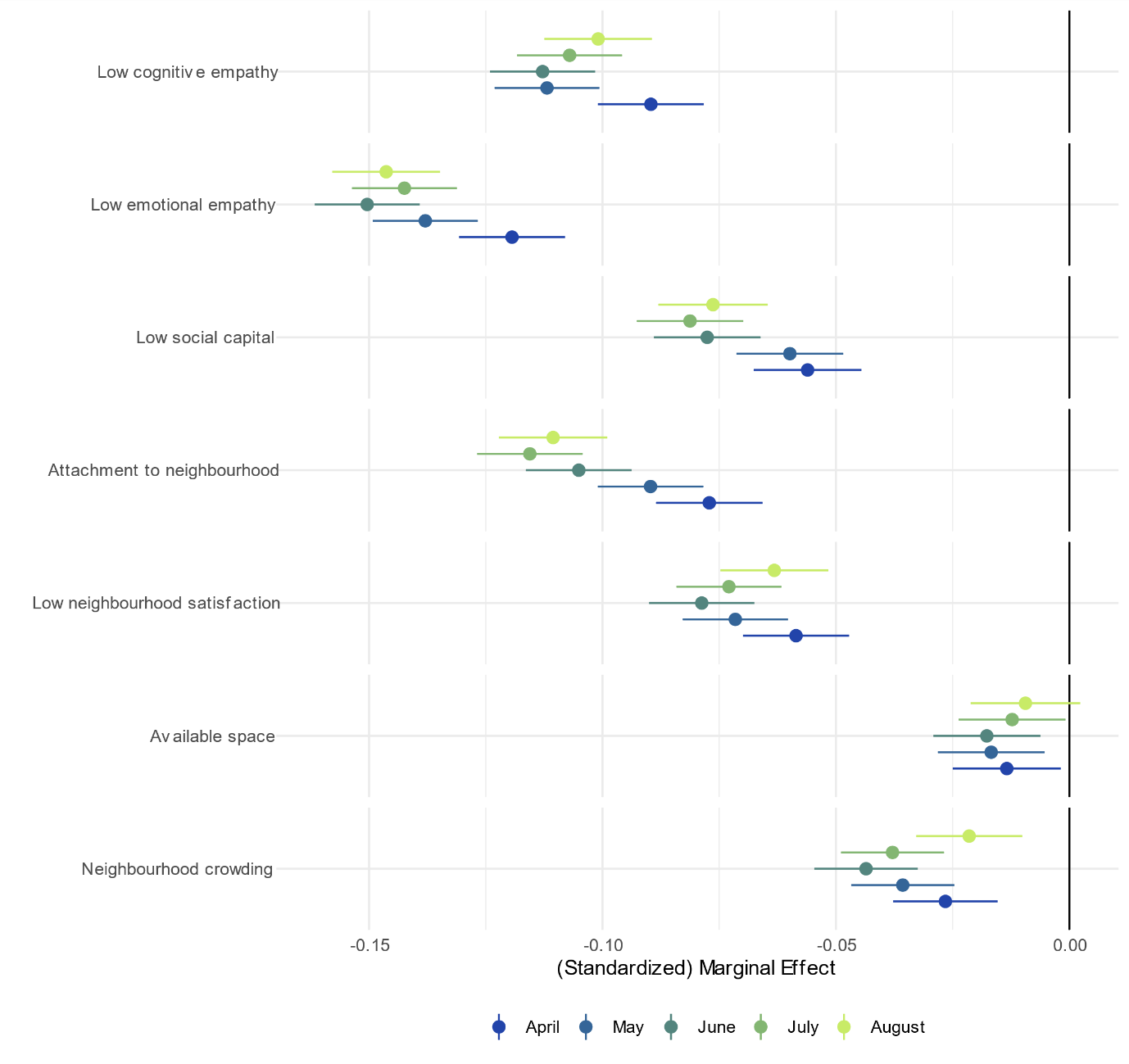


**Figure S6:** Association between social and pro-social factors and compliance with COVID-19 guidelines by month, derived from mixed effects models. Bivariate associations.


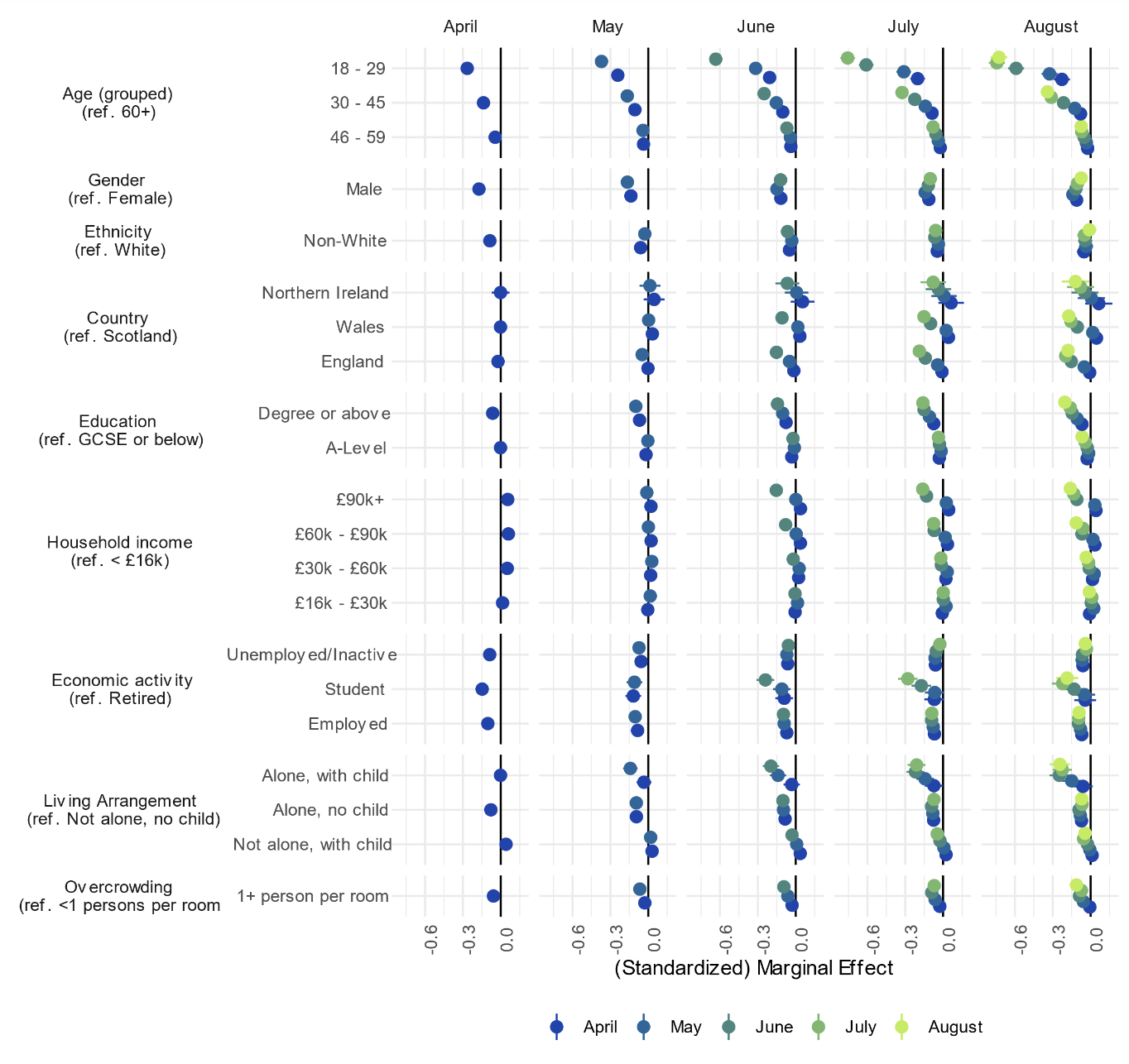


**Figure S7:** Association between demographic and socio-economic characteristics and compliance with COVID-19 guidelines by month, derived from mixed effects models. Each panel represents a different balanced panel of individuals interviewed up to a particular month. Models include adjustment for sex, age group, education, income group, employment status, country of residence, living arrangement, household overcrowding, whether the participant is shielding, diagnosed psychiatric condition, and Big-5 personality traits.


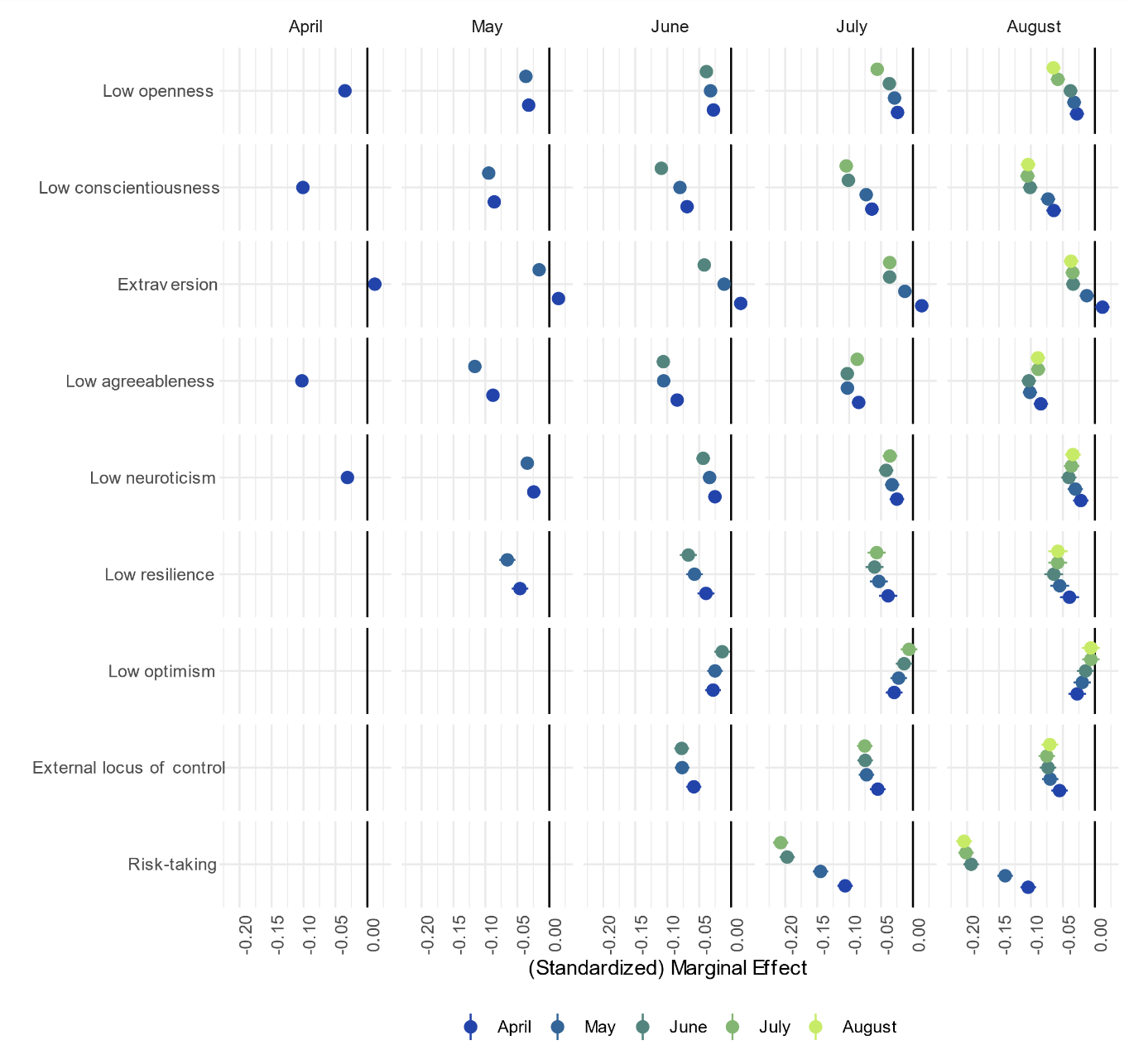


**Figure S8:** Association between personality traits and compliance with COVID-19 guidelines by month, derived from mixed effects models. Each panel represents a different balanced panel of individuals interviewed up to a particular month. Models include adjustment for sex, age group, education, income group, employment status, country of residence, living arrangement, household overcrowding, whether the participant is shielding, diagnosed psychiatric condition, and Big-5 personality traits.


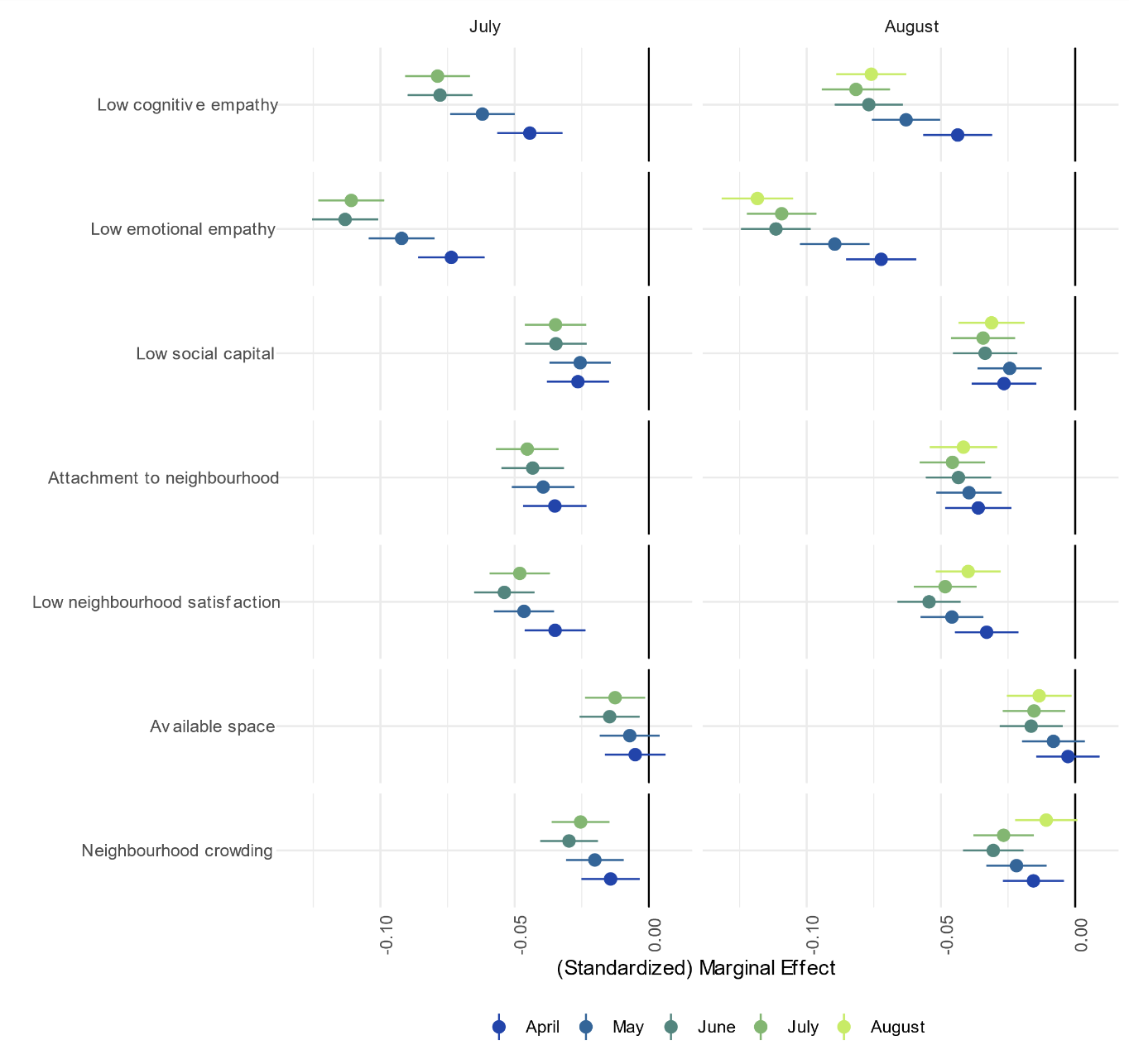


**Figure S9:** Association between social and pro-social factors and compliance with COVID-19 guidelines by month, derived from mixed effects models. Each panel represents a different balanced panel of individuals interviewed up to a particular month. Models include adjustment for sex, age group, education, income group, employment status, country of residence, living arrangement, household overcrowding, whether the participant is shielding, diagnosed psychiatric condition, and Big-5 personality traits.


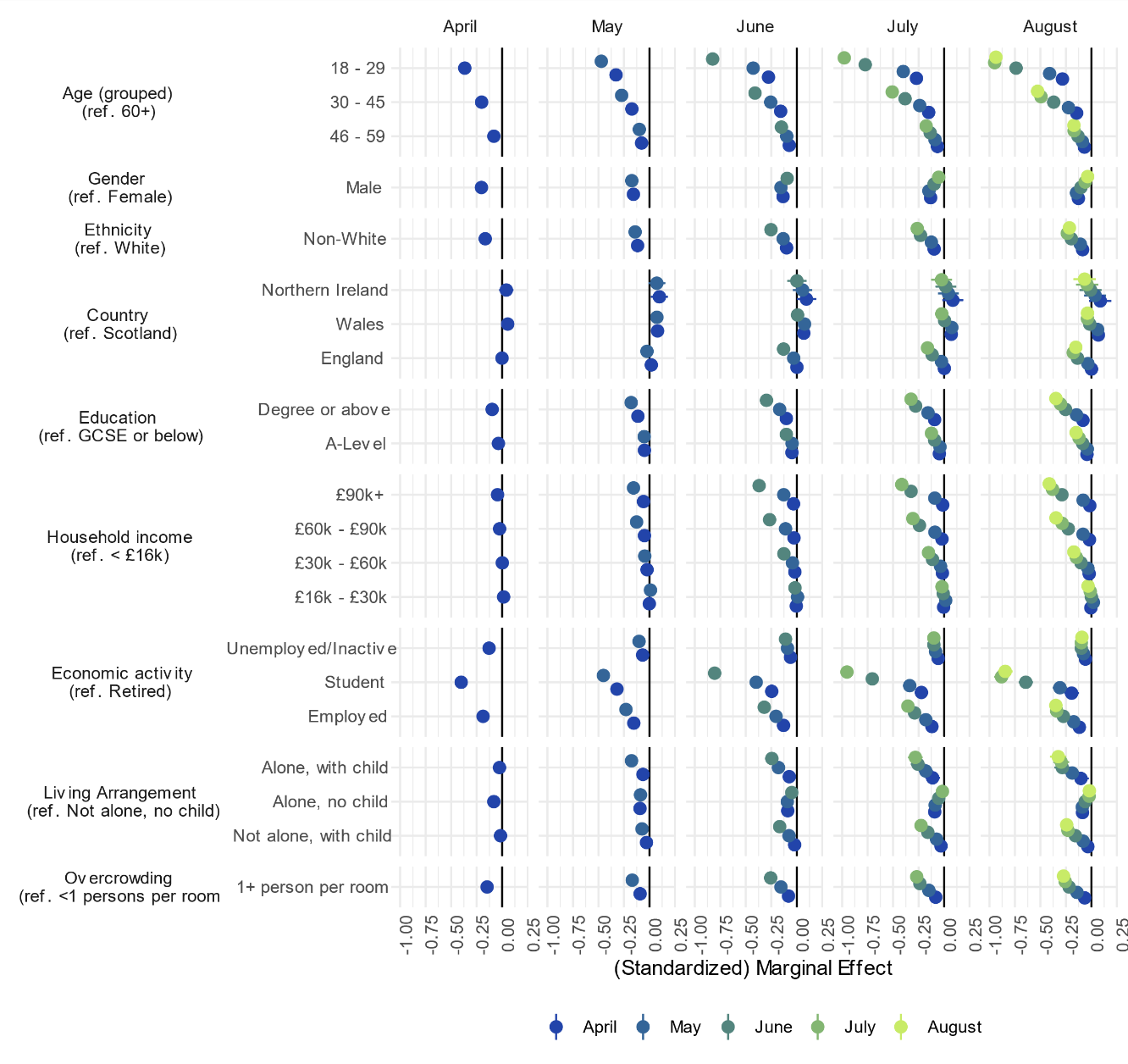


**Figure S10:** Association between demographic and socio-economic characteristics and compliance with COVID-19 guidelines by month, derived from mixed effects models. Each panel represents a different balanced panel of individuals interviewed up to a particular month. Bivariate associations.


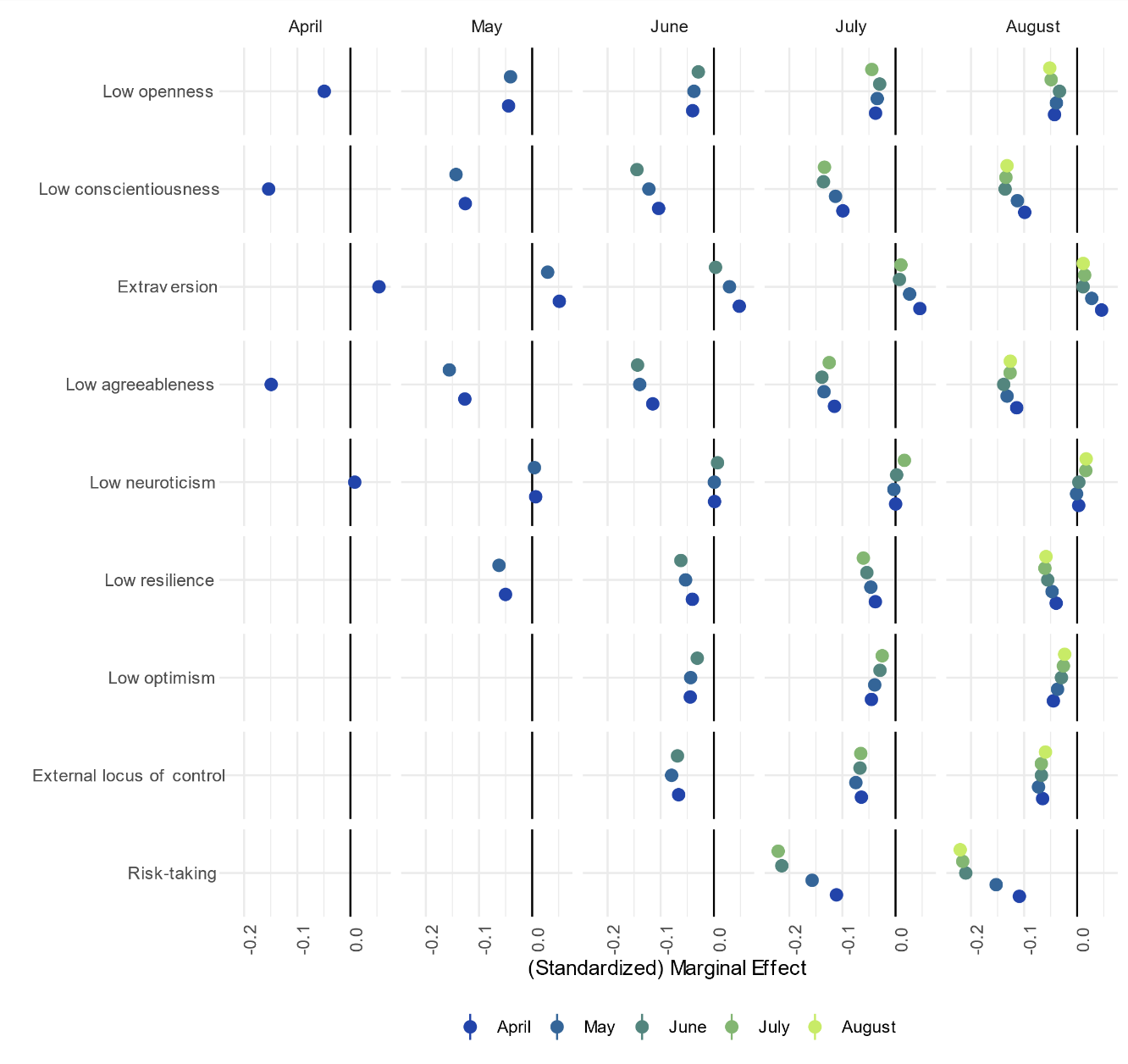


**Figure S11:** Association between personality traits and compliance with COVID-19 guidelines by month, derived from mixed effects models. Each panel represents a different balanced panel of individuals interviewed up to a particular month. Bivariate associations.


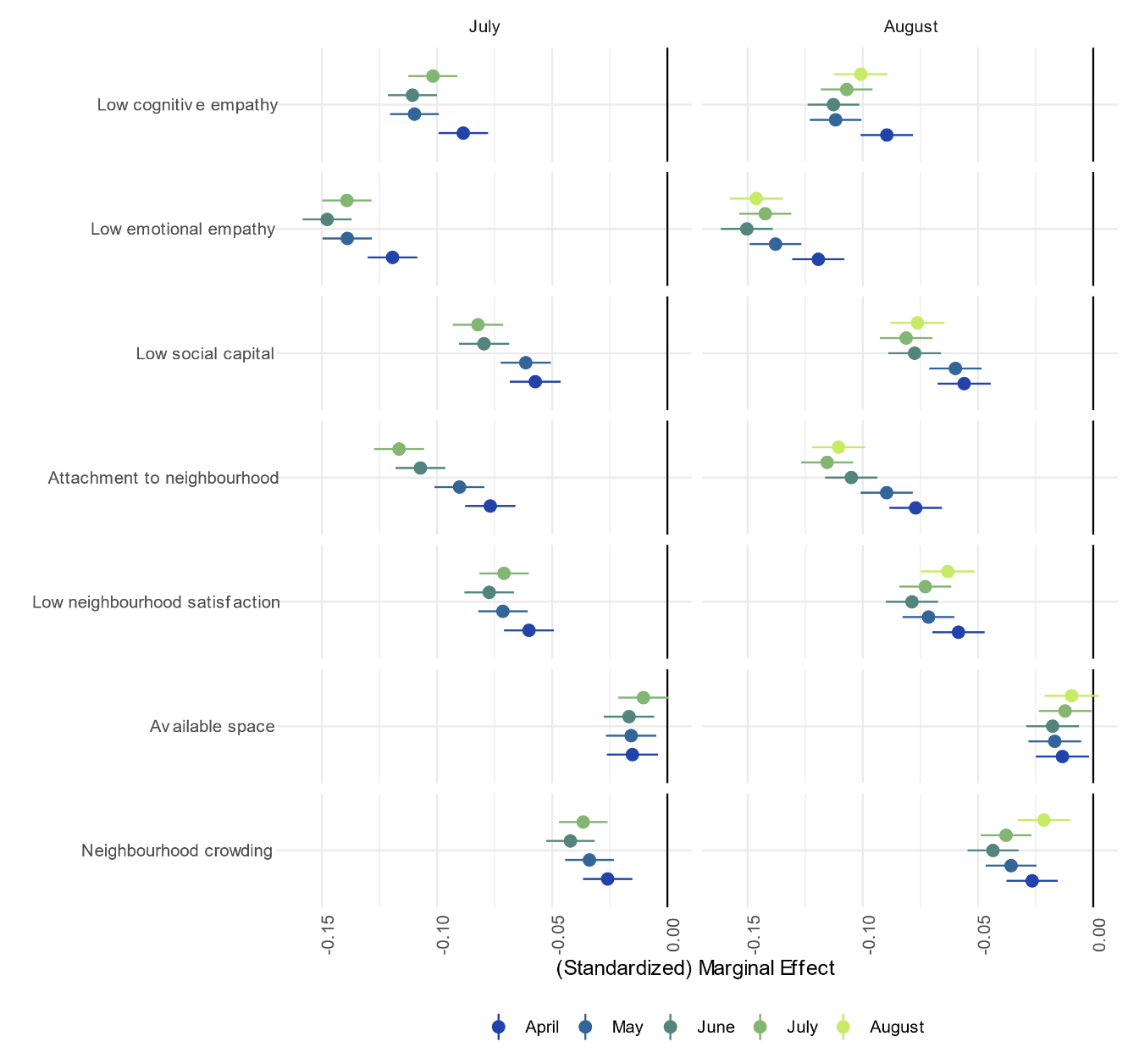


**Figure S12:** Association between social and pro-social factors and compliance with COVID-19 guidelines by month, derived from mixed effects models. Each panel represents a different balanced panel of individuals interviewed up to a particular month. Bivariate associations.


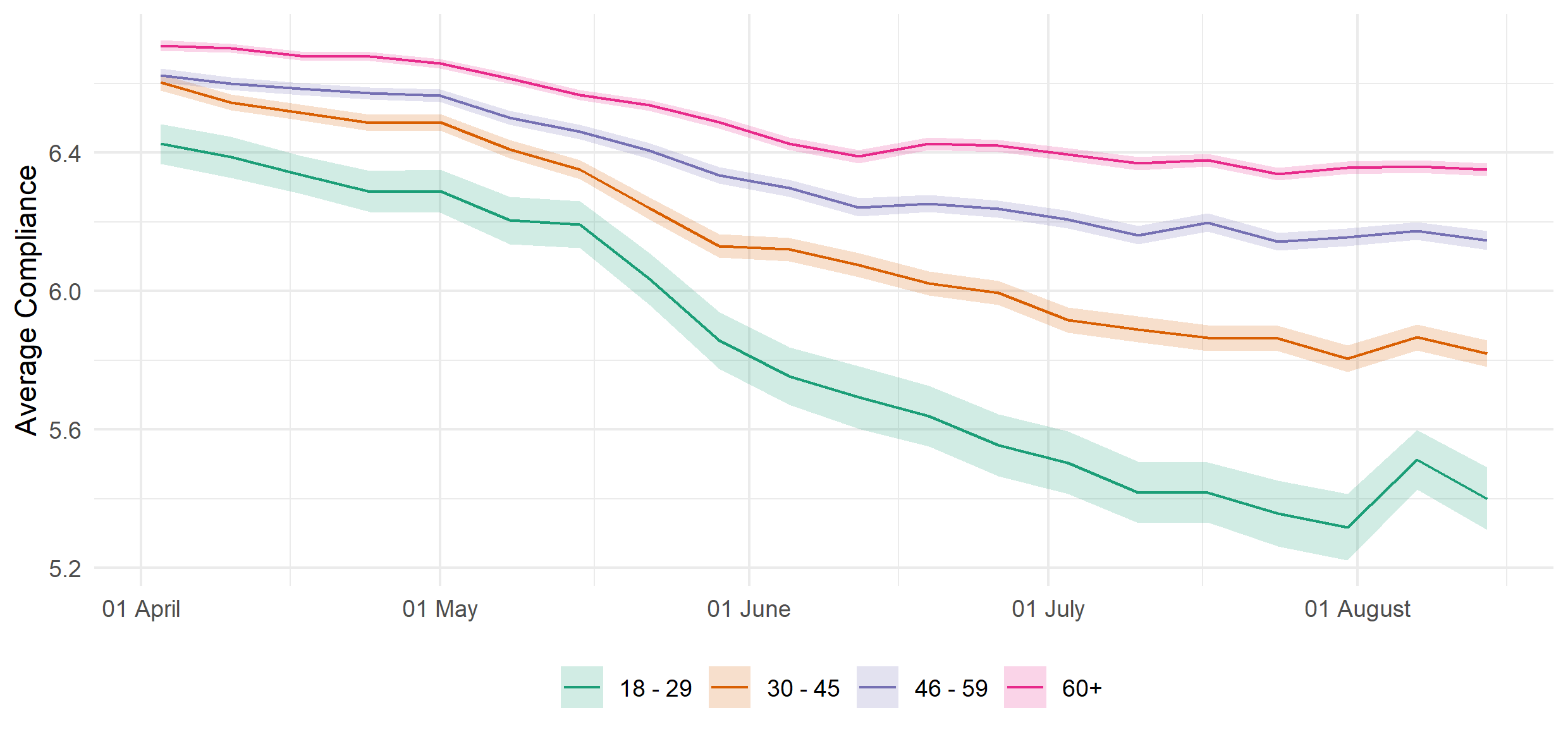


**Figure S13:** (Weighted) weekly average compliance by age group.


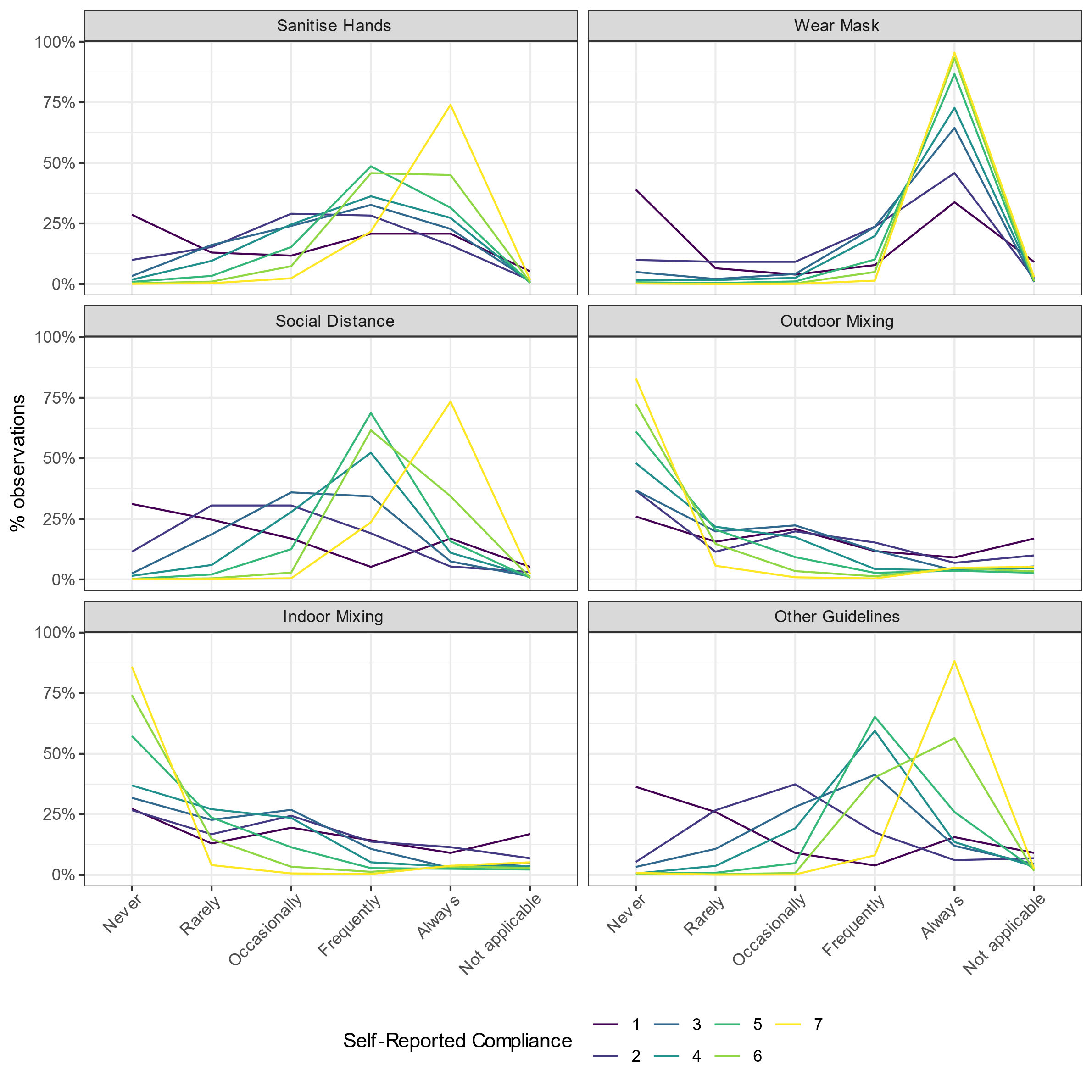


**Figure S14:** Distribution of responses to compliance behaviour items by self-reported compliance with guidelines level. N.B. outdoor mixing and indoor mixing asked about mixing with more than the permitted number of people, so answers reporting more frequent behaviours indicated lower compliance.


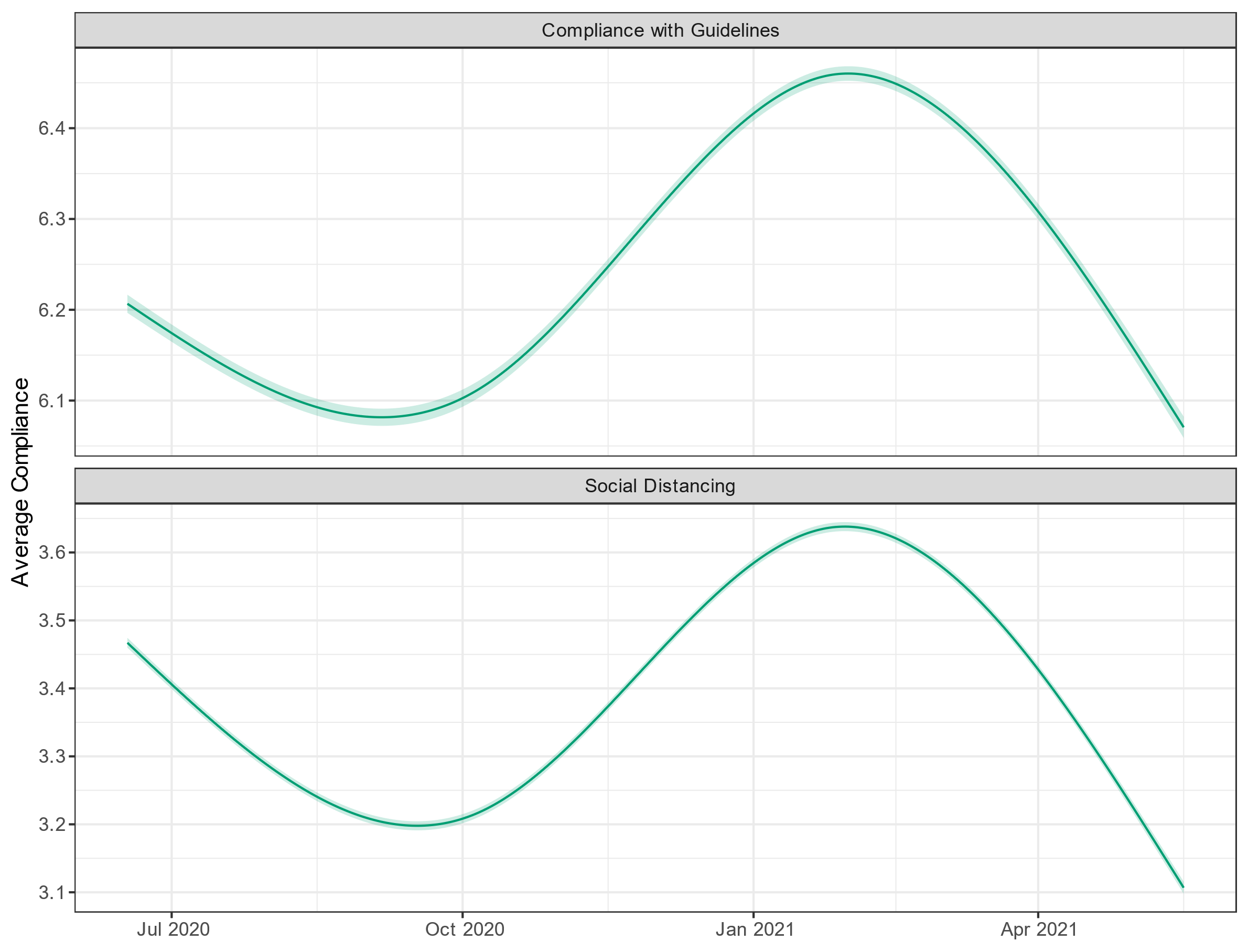


**Figure S15:** Estimated trends in self-reported compliance with guidelines and compliance with social distancing. Derived from growth curve models with time trends modelled using natural splines with 3 degrees of freedom. Growth curve models included random intercepts and slopes. Analysis sample restricted to participants with 3+ waves of data between 17 June 2020 – 17 May 2021.

## TABLES

**Table S1:** (Weighted) descriptive statistics by last month of interview. Participants interviewed in July are included in the main analysis. Participants last interviewed in earlier months are excluded.

|  | Variable | April | May | June | July | August |
| --- | --- | --- | --- | --- | --- | --- |
| Age (grouped) | 60+ | 2,726.05 (17.58%)* | 1,445.89 (22.49%)* | 812.85 (23.67%)* | 693.76 (29.86%)* | 11,679.69 (51.62%) |
|  | 46 - 59 | 3,424.15 (22.09%) | 1,661.85 (25.85%) | 986.11 (28.72%) | 668.49 (28.78%) | 5,799.04 (25.63%) |
|  | 30 - 45 | 4,972.34 (32.07%) | 1,949.66 (30.32%) | 1,038.56 (30.24%) | 625.01 (26.91%) | 3,632.15 (16.05%) |
|  | 18 - 29 | 4,381.46 (28.26%) | 1,372.60 (21.35%) | 596.48 (17.37%) | 335.74 (14.45%) | 1,514.13 (6.69%) |
| Gender | Female | 7,750.92 (49.99%) | 3,378.01 (52.54%)* | 1,897.1 (55.24%)* | 1,207.17 (51.97%)* | 11,111.33 (49.11%) |
|  | Male | 7,753.08 (50.01%) | 3,051.99 (47.46%) | 1,536.9 (44.76%) | 1,115.83 (48.03%) | 11,513.67 (50.89%) |
| Ethnicity | White | 13,033.28 (84.06%)* | 5,586.04 (86.87%)* | 2,985.94 (86.95%)* | 2,093.15 (90.11%)* | 21,116.39 (93.33%) |
|  | Non-White | 2,470.72 (15.94%) | 843.96 (13.13%) | 448.06 (13.05%) | 229.85 (9.89%) | 1,508.61 (6.67%) |
| Country | Scotland | 1,510.31 (9.74%)* | 585.92 (9.11%)* | 315.52 (9.19%)* | 210.26 (9.05%)* | 1,822.22 (8.05%) |
|  | England | 12,740.30 (82.17%) | 5,317.33 (82.7%) | 2,883.58 (83.97%) | 1,937.52 (83.41%) | 18,624.75 (82.32%) |
|  | Wales | 679.81 (4.38%) | 353.16 (5.49%) | 168.39 (4.9%) | 134.43 (5.79%) | 1,742.84 (7.7%) |
|  | Northern Ireland | 573.59 (3.7%) | 173.59 (2.7%) | 66.51 (1.94%) | 40.79 (1.76%) | 435.18 (1.92%) |
| Education | GCSE or below | 5,292.83 (34.14%)* | 1,825.85 (28.4%)* | 823.03 (23.97%)* | 638.66 (27.49%)* | 7,494.49 (33.12%) |
|  | A-Level | 5,596.35 (36.1%) | 2,245.35 (34.92%) | 1,175.81 (34.24%) | 701.28 (30.19%) | 7,271.22 (32.14%) |
|  | Degree or above | 4,614.82 (29.77%) | 2,358.80 (36.68%) | 1,435.16 (41.79%) | 983.06 (42.32%) | 7,859.29 (34.74%) |
| Household income | < £16k | 3,103.45 (22.23%)* | 1,259.01 (21.72%)* | 539.57 (17.25%)* | 398.58 (18.99%)* | 4,099.51 (20.3%) |
|  | £16k - £30k | 3,789.79 (27.15%) | 1,349.84 (23.28%) | 730.87 (23.37%) | 528.83 (25.19%) | 6,271.51 (31.06%) |
|  | £30k - £60k | 4,214.80 (30.2%) | 1,897.69 (32.73%) | 1,079.04 (34.5%) | 699.39 (33.32%) | 6,394.21 (31.67%) |
|  | £60k - £90k | 1,722.86 (12.34%) | 753.20 (12.99%) | 460.20 (14.72%) | 274.84 (13.09%) | 2,124.96 (10.52%) |
|  | £90k+ | 1,126.77 (8.07%) | 537.68 (9.27%) | 317.58 (10.16%) | 197.52 (9.41%) | 1,300.31 (6.44%) |
| Economic activity | Retired | 1,600.74 (10.32%)* | 891.04 (13.86%)* | 504.93 (14.7%)* | 463.52 (19.95%)* | 8,618.23 (38.09%) |
|  | Employed | 9,772.36 (63.03%) | 4,002.05 (62.24%) | 2,182.27 (63.55%) | 1,376.80 (59.27%) | 10,677.94 (47.2%) |
|  | Student | 1,601.99 (10.33%) | 573.77 (8.92%) | 284.89 (8.3%) | 169.50 (7.3%) | 785.86 (3.47%) |
|  | Unemployed/Inactive | 2,528.91 (16.31%) | 963.13 (14.98%) | 461.92 (13.45%) | 313.18 (13.48%) | 2,542.97 (11.24%) |
| Living Arrangement | Not alone, no child | 7,883.05 (50.85%)* | 3,340.47 (51.95%)* | 1,828.27 (53.24%)* | 1,310.06 (56.4%)* | 13,987.61 (61.82%) |
|  | Not alone, with child | 4,767.97 (30.75%) | 1,781.59 (27.71%) | 924.19 (26.91%) | 519.96 (22.38%) | 3,280.05 (14.5%) |
|  | Alone, no child | 2,280.52 (14.71%) | 1,133.35 (17.63%) | 585.89 (17.06%) | 422.49 (18.19%) | 5,014.07 (22.16%) |
|  | Alone, with child | 572.46 (3.69%) | 174.59 (2.72%) | 95.65 (2.79%) | 70.49 (3.03%) | 343.27 (1.52%) |
| Overcrowding | <1 persons per room | 11,188.74 (72.17%)* | 5,176.38 (80.5%)* | 2,903.78 (84.56%)* | 2,022.4 (87.06%)* | 20,616.22 (91.12%) |
|  | 1+ person per room | 4,315.26 (27.83%) | 1,253.62 (19.5%) | 530.22 (15.44%) | 300.6 (12.94%) | 2,008.78 (8.88%) |
|  | Low openness | 9.02 (3.51)* | 8.96 (3.43)* | 8.93 (3.35)* | 8.9 (3.3)* | 9.17 (3.25) |
|  | Low conscientiousness | 8.82 (3.32)* | 8.58 (3.15)* | 8.54 (3.16)* | 8.34 (3.13)* | 8.14 (2.94) |
|  | Extraversion | 12.92 (4.23)* | 12.58 (4.36) | 12.67 (4.37) | 12.74 (4.2) | 12.59 (4.25) |
|  | Low agreeableness | 8.65 (3.23)* | 8.71 (3.2)* | 8.69 (3.16) | 8.7 (3.14) | 8.58 (3.09) |
|  | Low neuroticism | 12.01 (4.54)* | 12.11 (4.55)* | 12.49 (4.42)* | 12.66 (4.38)* | 13.2 (4.33) |
|  | Low resilience | - | 16.62 (5.43)* | 16.17 (5.28)* | 16.11 (5.34)* | 15.35 (5.24) |
|  | Low optimism | - | - | 17.02 (4.69)* | 17.11 (4.77)* | 16.62 (4.7) |
|  | External locus of control | - | - | 12.56 (2.78) | 12.67 (2.81)* | 12.47 (2.69) |
| Shielding (pre-existing condition) | No | 13,145.35 (84.79%)* | 5,385.69 (83.76%)* | 2,837.38 (82.63%)* | 1,848.81 (79.59%)* | 17,263.31 (76.3%) |
|  | Yes | 2,358.65 (15.21%) | 1,044.31 (16.24%) | 596.62 (17.37%) | 474.19 (20.41%) | 5,361.69 (23.7%) |
| Shielding (family member) | No | 13,491.38 (87.02%)* | 5,611.88 (87.28%)* | 2,965.77 (86.36%) | 1,992.97 (85.79%) | 19,437.56 (85.91%) |
|  | Yes | 2,012.62 (12.98%) | 818.12 (12.72%) | 468.23 (13.64%) | 330.03 (14.21%) | 3,187.44 (14.09%) |
| Home for other reason | No | 15,006.66 (96.79%)* | 6,172.76 (96%)* | 3,289.87 (95.8%)* | 2,206.1 (94.97%) | 21,419.2 (94.67%) |
|  | Yes | 497.34 (3.21%) | 257.24 (4%) | 144.13 (4.2%) | 116.9 (5.03%) | 1,205.8 (5.33%) |
| Psychiatric condition | No | 11,879.4 (76.62%)* | 4,943.56 (76.88%)* | 2,744.92 (79.93%)* | 1,826.46 (78.63%)* | 19,203.27 (84.88%) |
|  | Yes | 3,624.6 (23.38%) | 1,486.44 (23.12%) | 689.08 (20.07%) | 496.54 (21.37%) | 3,421.73 (15.12%) |
| Long-Term Conditions | 0 | 9,799.83 (63.21%)* | 3,949.03 (61.42%)* | 2,161.99 (62.96%)* | 1,384.60 (59.6%)* | 11,660.97 (51.54%) |
|  | 1 | 3,642.38 (23.49%) | 1,568.74 (24.4%) | 779.53 (22.7%) | 527.88 (22.72%) | 6,493.88 (28.7%) |
|  | 2+ | 2,061.79 (13.3%) | 912.24 (14.19%) | 492.48 (14.34%) | 410.53 (17.67%) | 4,470.15 (19.76%) |

# References

Brouard, S., Vasilopoulos, P., & Becher, M. (2020). Sociodemographic and Psychological Correlates of Compliance with the COVID-19 Public Health Measures in France. *Canadian Journal of Political Science*, 1–6. https://doi.org/10.1017/S0008423920000335

Clark, C., Davila, A., Regis, M., & Kraus, S. (2020). Predictors of COVID-19 voluntary compliance behaviors: An international investigation. *Global Transitions*, *2*, 76–82. https://doi.org/10.1016/j.glt.2020.06.003

Davis, M. H. (1983). Measuring individual differences in empathy: Evidence for a multidimensional approach. *Journal of Personality and Social Psychology*, *44*(1), 113–126. https://doi.org/10.1037/0022-3514.44.1.113

Dohmen, T., Falk, A., Huffman, D., Sunde, U., Schupp, J., & Wagner, G. G. (2011). INDIVIDUAL RISK ATTITUDES: MEASUREMENT, DETERMINANTS, AND BEHAVIORAL CONSEQUENCES. *Journal of the European Economic Association*, *9*(3), 522–550. https://doi.org/10.1111/j.1542-4774.2011.01015.x

Hale, T., Angrist, N., Cameron-Blake, E., Hallas, L., Kira, B., Majumdar, S., Petherick, A., Phillips, T., Tatlow, H., & Webster, S. (2020). *Oxford COVID-19 Government Response Tracker*. Blavatnik School of Government. https://www.bsg.ox.ac.uk/research/research-projects/coronavirus-government-response-tracker

Hirschman, C., & Almgren, G. (2012). *University of Washington - Beyond High School (UW-BHS): Version 5* [Data set]. ICPSR - Interuniversity Consortium for Political and Social Research. https://doi.org/10.3886/ICPSR33321.V5

Mujahid, M. S., Diez Roux, A. V., Morenoff, J. D., & Raghunathan, T. (2007). Assessing the Measurement Properties of Neighborhood Scales: From Psychometrics to Ecometrics. *American Journal of Epidemiology*, *165*(8), 858–867. https://doi.org/10.1093/aje/kwm040

Peck, C. A. (1981). *Relationship between satisfaction with housing and quality of life* [PhD Thesis, Oklahoma State University]. https://core.ac.uk/download/pdf/215251197.pdf

Scheier, M. F., Carver, C. S., & Bridges, M. W. (1994). Distinguishing optimism from neuroticism (and trait anxiety, self-mastery, and self-esteem): A reevaluation of the Life Orientation Test. *Journal of Personality and Social Psychology*, *67*(6), 1063–1078. https://doi.org/10.1037/0022-3514.67.6.1063

Smith, B. W., Dalen, J., Wiggins, K., Tooley, E., Christopher, P., & Bernard, J. (2008). The brief resilience scale: Assessing the ability to bounce back. *International Journal of Behavioral Medicine*, *15*(3), 194–200. https://doi.org/10.1080/10705500802222972

Soto, C. J., & John, O. P. (2017). The next Big Five Inventory (BFI-2): Developing and assessing a hierarchical model with 15 facets to enhance bandwidth, fidelity, and predictive power. *Journal of Personality and Social Psychology*, *113*(1), 117–143. https://doi.org/10.1037/pspp0000096

YouGov. (2021). *Personal measures taken to avoid COVID-19*. https://yougov.co.uk/topics/international/articles-reports/2020/03/17/personal-measures-taken-avoid-covid-19

Zajenkowski, M., Jonason, P. K., Leniarska, M., & Kozakiewicz, Z. (2020). Who complies with the restrictions to reduce the spread of COVID-19?: Personality and perceptions of the COVID-19 situation. *Personality and Individual Differences*, *166*, 110199. https://doi.org/10.1016/j.paid.2020.110199
